# A medium-entropy transition metal oxide cathode for high-capacity lithium metal batteries

**DOI:** 10.1038/s41467-022-33927-0

**Published:** 2022-10-18

**Authors:** Yi Pei, Qing Chen, Meiyu Wang, Pengjun Zhang, Qingyong Ren, Jingkai Qin, Penghao Xiao, Li Song, Yu Chen, Wen Yin, Xin Tong, Liang Zhen, Peng Wang, Cheng-Yan Xu

**Affiliations:** 1 Sauvage Laboratory for Smart Materials, School of Materials Science and Engineering, Harbin Institute of Technology (Shenzhen), Shenzhen, 518055 China; 2 National Laboratory of Solid State Microstructures, College of Engineering and Applied Sciences, Collaborative Innovation Center of Advanced Microstructures and Center for the Microstructures of Quantum Materials, Nanjing University, Nanjing, 210093 China; 3 National Synchrotron Radiation Laboratory, CAS Center for Excellence in Nanoscience, University of Science and Technology of China, Hefei, 230029 China; 4 Institute of High Energy Physics, Chinese Academy of Sciences, Beijing, 100049 China; 5 Spallation Neutron Source Science Center, Dongguan, 523803 China; 6 Department of Physics & Atmospheric Science, Dalhousie University, Halifax, NS B3H 4R2 Canada; 7 School of Materials Science and Engineering, and MOE Key Laboratory of Micro-Systems and Micro-Structures Manufacturing, Harbin Institute of Technology, Harbin, 150001 China; 8 Department of Physics, University of Warwick, Coventry, CV4 7AL UK

**Keywords:** Batteries, Energy, Inorganic chemistry, X-ray diffraction, Materials for energy and catalysis

## Abstract

The limited capacity of the positive electrode active material in non-aqueous rechargeable lithium-based batteries acts as a stumbling block for developing high-energy storage devices. Although lithium transition metal oxides are high-capacity electrochemical active materials, the structural instability at high cell voltages (e.g., >4.3 V) detrimentally affects the battery performance. Here, to circumvent this issue, we propose a Li_1.46_Ni_0.32_Mn_1.2_O_4-*x*_ (0 < *x* < 4) material capable of forming a medium-entropy state spinel phase with partial cation disordering after initial delithiation. Via physicochemical measurements and theoretical calculations, we demonstrate the structural disorder in delithiated Li_1.46_Ni_0.32_Mn_1.2_O_4-*x*_, the direct shuttling of Li ions from octahedral sites to the spinel structure and the charge-compensation Mn^3+^/Mn^4+^ cationic redox mechanism after the initial delithiation. When tested in a coin cell configuration in combination with a Li metal anode and a LiPF_6_-based non-aqueous electrolyte, the Li_1.46_Ni_0.32_Mn_1.2_O_4-*x*_-based positive electrode enables a discharge capacity of 314.1 mA h g^−1^ at 100 mA g^−1^ with an average cell discharge voltage of about 3.2 V at 25 ± 5 °C, which results in a calculated initial specific energy of 999.3 Wh kg^−1^ (based on mass of positive electrode’s active material).

## Abstract (web summary)

Structural instability is a major drawback of high-capacity lithium-based battery cathodes. Here, the authors report a cathode active material with a medium-entropy state created by partial cation disordering capable of restraining the structural evolution in the high-capacity operated spinel phase.

## Introduction

**Fig. 1:**
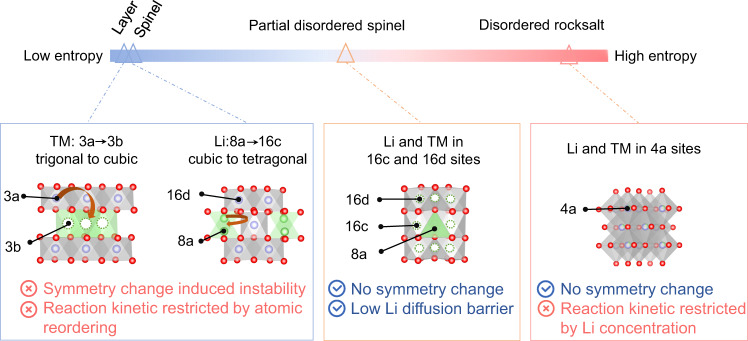
Schematic illustrations of the cation reordering and symmetry change upon lithiation/delithiation within different Li_*x*_TM_*y*_O_*2*_ phases. The 3a, 16c, 16d, and 4a octahedral sites are represented by gray octahedrons, while the 3b octahedral sites and 8a tetrahedral sites are shown as green octahedrons and tetrahedrons, respectively. Transition metal ions, Li ions, and oxygen ions are shown as purple, green, and red balls, respectively, and the probable occupancies of vacancies are marked as green dashed cycles.

The development of Li-ion batteries (LIBs) has recently motivated innovation moving from internal combustion engine vehicles toward battery electric vehicles (BEVs), and the requirement to further improve the cruising distance of BEVs calls for cathode materials with higher energy/power densities^1–3^. Although 3*d* transition metal (TM)-based lithium transition metal oxides (Li_*x*_TM_*y*_O_2_, 0 < *x*, *y* < 2, TM = Ni, Mn, Co, etc) can deliver a theoretical capacity of >270 mA h g^−1^, such high-capacity operation triggers the migration of TM or Li ions and brings about structural (e.g., symmetry) changes (Fig. 1)^4–8^. Therefore, the high-capacity operation of Li_*x*_TM_*y*_O_2_ is always accompanied by continuous phase evolution, such as layer to spinel, layer to rocksalt, and spinel to T1/T2 phase transitions in high-capacity operated (>200 mA h g^−1^) LiCoO_2_^9^, LiNi_*a*_Mn_*b*_Co_*c*_O_2_ (*a* + *b* + *c* = 1)^10,11^, Li_1+*x*_TM_1-*x*_O_2_ (0 < *x* < 1)^12–14^, and LiNi_0.5_Mn_1.5_O_4_^15^, resulting in unsatisfactory cycling stability and rate performance in most high-capacity cathodes. Consequently, a critical direction in the establishment of a high-energy density cathode is the prevention of the continuous reordering of cations upon high-capacity operation of Li_*x*_TM_*y*_O_2_.

The symmetry changes of Li_*x*_TM_*y*_O_2_ have been revealed to be driven by the shift in site energies under different states^16–18^, and the thermodynamically stable phase of Li_*x*_TM_*y*_O_2_ polytypes generally varies with the Li concentration (Supplementary Fig. 1). For most 3*d* TM-based Li_*x*_TM_*y*_O_2_ with cation ordering, namely, low-entropy state phases, such cation disordering is thermodynamically inevitable during high-capacity operation (>200 mA h g^−1^)^7,12,19–21^. Recently, specific discharge capacity values higher than 300 mA h g^−1^ (at low specific currents, e.g., <20 mA g^−1^, and for a very limited number of cycles) are reported in the literature using disordered rocksalt (DRX) cathodes and applying a low cutoff voltage of 1.5 V^22^. In these materials, all the cations are randomly distributed in the 4a sites to form a higher entropy state than that of cation-ordered Li_*x*_TM_*y*_O_2_. The principle of entropy increase prevents the reordering of cations in DRX, thus effectively avoiding symmetry changes upon high-capacity operation (>200 mA h g^−1^)^23–25^. However, the thermodynamic trend of cation disordering is found to be strongly associated with the type of TM ion^26,27^ and chemical composition^28,29^, while the reaction kinetics depend on the synthesis conditions. Our calculations also indicate that cation disordering is thermodynamically unfavorable in 3*d* TM-based Li_*x*_Ni_0.25_Mn_0.75_O_2_ (0 < *x* < 1, Supplementary Fig. 1). Moreover, although DRX with rapid Li^+^ diffusion kinetics could be achieved through tailored synthetic strategy^30,31^, Li^+^ diffusion through the percolation path in these high-entropy state DRX phases is restricted by the Li^+^ concentration and local conditions^32^, resulting in a great challenge in achieving suitable DRX towards practical application^25,33,34^.

In this work, we demonstrate that the high-capacity operation of Li_*x*_TM_*y*_O_2_ (0 < *x*, *y* < 2, TM = Ni, Mn) could be achieved by establishing a partial cation-disordered medium-entropy state. To break the thermodynamically driven TM ion ordering, we prepared the defective Li_1.46_Ni_0.32_Mn_1.2_O_4–*x*_ (0 < *x* < 4) through proton substitution, further triggering the interlayer disordering of TM ions to form a partial cation-disordered spinel phase upon initial delithiation. This partial cation-disordered spinel crystal, which has only been reported in oxyfluorides^35,36^, was confirmed by high-angle annular dark-field scanning transmission electron microscopy (HAADF-STEM) and synchrotron X-ray diffraction (SXRD) measurements, distinguishing itself from LiTM_2_O_4_-type spinel phases by TM ion filling of both the 16c and 16d interstitial sites. When tested in a coin cell configuration in combination with a Li-metal anode and a LiPF_6_-based non-aqueous electrolyte, the medium-entropy state cathode material enabled a reversible capacity of 314.1 mA h g^−1^ at 100 mA g^−1^ (2.0–4.8 V, 25 ± 5 °C) and mitigated structural degradation, and the spinel framework ensured Li^+^ diffusion under elevated currents (discharge capacity of 153.6 mA h g^−1^ under 1 A g^−1^). Combining electrochemical characterization, neutron diffraction (ND) measurements, and DFT calculations, we found that Li ions are mostly shuttled from the octahedral sites, therefore circumventing the continuous symmetry change in the well-ordered layered and spinel phases. X-ray photoelectron spectroscopy (XPS), X-ray absorption spectroscopy (XAS), and operando differential electrochemical mass spectrometry (DEMS) results revealed the oxidation of O^2−^ during initial charging, while the discharging process was primarily charge compensated by Mn^3+^/Mn^4+^ redox center.

## Results

### Long- and short-term atomic ordering of defective Li_1.46_Ni_0.32_Mn_1.2_O_4–*x*_

**Fig. 2:**
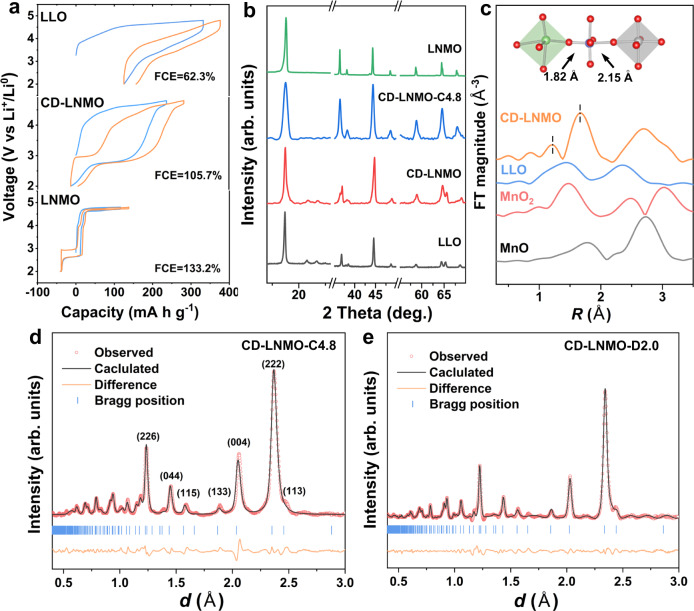
Electrochemical and structural characterization of different lithium transition metal oxide materials. **a** Comparison of the first two cycle voltage profiles of LLO, CD-LNMO, and LNMO from 2.0–4.8 V. The tests were performed with a specific current of 100 mA g^−1^ at 25 ± 5 °C. **b** Synchrotron X-ray (SXRD) patterns of CD-LNMO and fully charged CD-LNMO (denoted as CD-LNMO-C4.8). The laboratory XRD patterns of LLO and LNMO are shown as references. Both the synchrotron and laboratory X-ray measurements were performed under the wavelength of Cu radiator (1.54 Å). **c** ***k***^3^-weighted Fourier transform magnitudes of Mn K-edge EXAFS spectra obtained from LLO and CD-LNMO. Mn K-edge EXAFS spectra of MnO and MnO_2_ are shown as references. The short and long first-shell coordination peaks (within 1~2 Å) of CD-LNMO could be assigned to the intensified MnO_6_ distortion near the cation-disordered regions. The bond lengths of MnO near the cation-disordered region were determined from the DFT calculations of LLO with partial cation disordering (Supplementary Figure 14). **d**, **e** Time-of-flight neutron diffraction (ND) data of CD-LNMO electrodes under 4.8 V charged (**d**) and 2.0 V discharged (**e**) states in the initial cycle. The weakening of the (004) reflections after lithiation implies the insertion of Li ions into the octahedral sites (Supplementary Fig. 12 and Supplementary Note 2). The goodness-of-fit parameters (*R*_wp_) are 2.78% and 4.86%, respectively.

The defective Li_1.46_Ni_0.32_Mn_1.2_O_4–*x*_ (0 < *x* < 4) cathode (CD-LNMO) was prepared by proton exchange followed by manipulated cation reordering in Li-rich layered oxides (layered-type: Li_1.2_Ni_0.2_Mn_0.6_O_2_, denoted as LLO, details are shown in the Methods section and Supplementary Note 1, Phase evolution details shown in Supplementary Figs. 2–4), while the untreated LLO, commercial spinel oxides (spine-type: LiNi_0.5_Mn_1.5_O_4_, denoted as LNMO) were also tested for comparison. Transmission electron microscopy (TEM) images revealed a nanosheet morphology of the CD-LNMO (Supplementary Fig. 5), and the chemical composition, mostly the ratio of Li:Mn:Ni of CD-LNMO, was confirmed by inductively coupled plasma–mass spectrometry (ICP–MS). As shown in Supplementary Table 1, the decreased Ni and Li contents in CD-LNMO suggest the preferential substitution of Ni and Li by protons, most likely associated with the lower binding energies of Li^+^-O^2−^ and Ni^2+^-O^2−^ than that of Mn^4+^-O^2−^. We, therefore, define the stoichiometric ratio of O as 4–*x* to accommodate the uncertainty arising from the probable valence change of TM ions. Interestingly, even though the Li:TM ratio in CD-LNMO is situated between LLO and LNMO, the electrochemical behavior of CD-LNMO (Fig. 2a) was different from that of the LLO/LNMO composites in previous reports^37–44^. After the electrochemical removal of Li ions during the initial charging process, CD-LNMO showed two discharge plateaus at ~4.6 V and ~2.7 V, which are associated with the insertion of Li ions into tetrahedral sites and octahedral sites in the spinel-type structure, respectively^45^. Generally, for LNMO, LiMn_2_O_4_, and Li_4_Mn_5_O_12_ with ideal spinel-type ordering (full-occupied 16d sites and empty 16c sites), the inserted Li ions will first occupy all of the 8a tetrahedral sites (discharge plateaus of ~4.6 or 4.0 V) and then migrate into the neighboring 16c octahedral sites (discharge plateau of ~2.7 V) concurrent with the rapid structural degradation caused by the cubic to tetragonal symmetry change^45–48^. However, we found a much longer 2.7 V plateau than that of 4.6 V in the discharge profile of CD-LNMO, implying that the majority of Li ions are directly inserted into the octahedral sites of delithiated CD-LNMO. Note that this feature also distinguishes it from the *I*41 or Li_*x*_Mn_3_O_4_ phases formed during the structural degradation of Li-rich layered oxides^32^, as the latter phase undergo the solid-solution or conversion reactions without a plateau at ~2.7 V^21,49^. In the following cycles, CD-LNMO shows a noticeable voltage hysteresis that is constantly observed in high-capacity cathodes^50–52^. Based on previous literature, this hysteresis is likely to suggest different reaction kinetics between the charge and discharge processes^34,35^. The charge–discharge profile of high temperature-treated Li_1.46_Ni_0.32_Mn_1.2_O_4–*x*_ (high temperature applied during synthesis, denoted as CD-LNMO-H) with more thorough cation reordering was investigated to reveal the correlation between the defect state and electrochemical behavior (Supplementary Fig. 6), from which a higher ratio of Li ions inserted into the tetrahedral sites than that of CD-LNMO. Therefore, it can be concluded that the defective state of as-synthesized CD-LNMO changes the Li-ion storage mechanism.

The evolution of long-term cation ordering in CD-LNMO during the first cycle was explored through ex situ SXRD, and the XRD patterns of LLO and LNMO are shown as references for the layered-type and spinel-type ordering of TM ions. As shown in Fig. 2b, the as-synthesized CD-LNMO is a layered/spinel composite, while the reflections of the fully delithiated sample (charged to 4.8 V) shift toward pure spinel-type ordering without impurity phases (Supplementary Figs. 7 and 8). Such a trend implies localized reordering during the removal of Li ions, and the diminished layered phase is considered indicative of TM ion migration. The fully lithiated sample (discharged to 2.0 V) retains the spinel-type ordering without the formation of the T1/T2 phase (Supplementary Figs. 7 and 9, and Supplementary Note 2)^15,40^, confirming the suppressed cubic to tetragonal symmetry change in conventional LNMO. Moreover, compared to the conventional LNMO, we found a weakened (111) reflection in the cycled electrodes (Fig. 2b and Supplementary Fig. 7), implying partial cation disorder in the cycled CD-LNMO (Supplementary Fig. 10, Supplementary Note 2)^35,36^. This was further shown by Rietveld refinements on the fully delithiated and lithiated samples, which revealed that ~15 at% and ~19 at% of the TM ions were located in the 16c site under 4.8 V and 2.0 V, respectively (Supplementary Table 2).

We further conducted Mn/Ni-K-edge extended X-ray absorption fine structure (EXAFS) measurements on the as-synthesized CD-LNMO and LLO to detect the short-term ordering of cations. Compared to the Mn and Ni-K-edge EXAFS spectra of LLO with a single Mn/Ni-O scattering path in the first shell (~1.45 Å for MnO and ~1.58 Å for Ni-O), the coexistence of two Mn/Ni-O bonds in the EXAFS spectra of as-synthesized CD-LNMO (Fig. 2c and Supplementary Fig. 11) implied an altered localized configuration of TM ions after the creation of defects. It is worth mentioning that such a short-term configuration is unlikely to be traced from the distinction of TM-O bonds in layered and spinel structures^2,20,53–55^, and the MnO bond length difference (~0.4 Å) is larger than that between the Mn^4+^-O^2−^ bonds in MnO_2_ and the Mn^2+^-O^2−^ bond in MnO. Instead, such a configuration suggests the breaking of Mn/NiO_6_ ordering^56^, which may be associated with the highly defective state in CD-LNMO. Our DFT calculations comparing the MnO bond lengths in a Li_1.2_Ni_0.2_Mn_0.6_O_2_ crystal with partial interlayer Li/TM mixing further supported this result (Supplementary Fig. 12). As shown in the inset of Fig. 2c, we found that the occupancy of TM ions in the Li layer resulted in substantial distortion of the adjacent MnO_6_ ligand traced from the much stronger TM-TM Coulombic repulsion than that of Li-TM, giving rise to an increased MnO bond length deviation (0.33 Å) compared to that in Li_1.2_Ni_0.2_Mn_0.6_O_2_ without Li/TM mixing (0.19 Å). Therefore, it is likely that breaking TMO_6_ ordering will aggravate the distortion of TMO_6_ ligands and promote the splitting of the TM-O scattering peak.

Considering the low scattering factor (*f*) of Li under X-ray measurements (*f*_Li_ < 2.0), ND measurements were performed to probe the occupancy of Li ions undercharged and discharged states (Fig. 2d, e). The TM ion occupancies obtained from the refinement of the SXRD patterns were adopted for the ND refinements, and the occupancies of Li ions in the 4.8 V charged and 2.0 V discharged samples are listed in Supplementary Table 2. The refinement revealed negligible Li-ion content in the 4.8 V charged sample, while most of the Li ions were inserted into the octahedral sites in the 2.0 V discharged sample (Supplementary Note 2, Supplementary Figs. 13 and 14, Supplementary Table 3). Therefore, we can conclude that delithiation of CD-LNMO promotes the formation of a medium-entropy state spinel phase with partial cation disordering, within which the Li ions are inserted/extracted primarily from the octahedral sites.

**Fig. 3:**
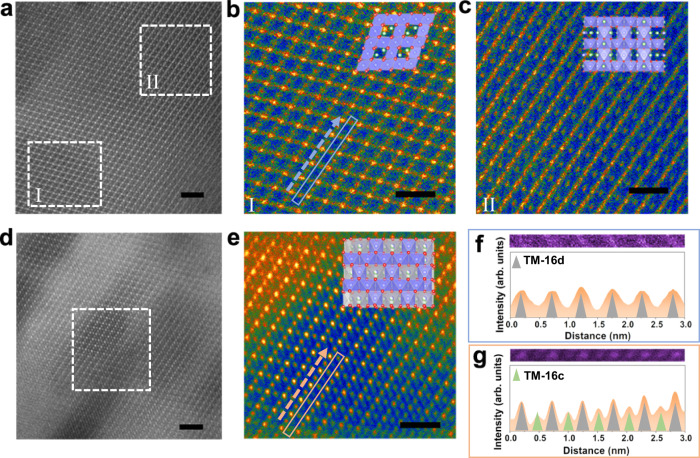
Ex situ STEM investigations of Li1.46Ni0.32Mn1.2O_4–x_-based electrodes. a–c. HADDF-STEM images of the as-synthesized CD-LNMO powder (**a**), and FFT-filtered images of the I (**b**), and II (**c**) regions. **d**, **e** HADDF-STEM images of the fully charged CD-LNMO powder. **e** FFT-filtered images of the marked region in **d**. **f**, **g** Enlarged HADDF-STEM images and corresponding signal profiles of the regions marked with blue and orange boxes in **b** and **e**, respectively. The occupancies of TM ions in the 16d and 16c sites are represented by gray and green triangles, respectively. Scale bars in **a**, **d** and **b**, **c**, **e** represent 2 and 1 nm, respectively.

TEM and HAADF-STEM images of the as-synthesized CD-LNMO display the coexistence of two regions with different contrasts (Fig. 3a–c). The FFT-filtered HAADF-STEM image revealed a 3D spinel-type ordering in dispersive low-contrast regions, while the high-contrast regions were likely to retain a disordered layer phase with partial TM ion disordering. This disordered layer phase was previously observed as an intermediate phase during the 2D to 3D reordering of layered oxides^57^, indicating cation disordering in CD-LNMO. An electron energy loss spectroscopy (EELS) scan witnessed the lower valence state of Mn in the spinel phase regions (Supplementary Fig. 15), which is consistent with the lower valence state of Mn in the nonstoichiometric spinel phase^14,58^. The removal of Li ions from CD-LNMO during the charging process facilitated the 3D ordering of TM ions (Fig. 3d). As shown in Fig. 3e–g, detailed HAADF-STEM signal profiles showcase the occupancies of TM ions in both the 16c and 16d sites of the spinel-type framework, confirming the partially disordered spinel structure after delithiation. The highly defective state of the as-synthesized CD-LNMO promotes partial cation disordering during the initial delithiation, and this process is highly dependent on the degree of cation ordering in CD-LNMO. Combining the electrochemical and XRD/ND characterizations, we can speculate that the medium-entropy state of delithiated CD-LNMO with partial cation disordering altered the Li-ion storage mechanism in the following cycles, while the majority of the Li ions were shuttled from the octahedral sites of the medium-entropy state phase.

### Electrochemical characterization of defective Li_1.46_Ni_0.32_Mn_1.2_O_4–*x*_

**Fig. 4:**
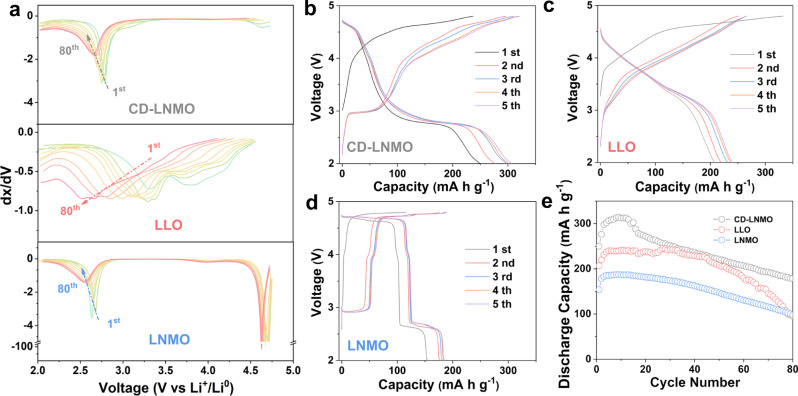
Electrochemical performance of different rocksalt-type transition metal oxide materials. **a** Differential capacity curves of CD-LNMO, LLO, and LNMO from the 1st to 80th cycles. The cycling tests were performed from 2.0–4.8 V at a specific current of 100 mA g^−1^, and the curves were plotted every 10 cycles. **b**–**d** The charge–discharge profile of CD-LNMO (**b**), LLO (**c**), and LNMO (**d**) in the first five cycles with a specific current of 100 mA g^−1^. **e** Cycling tests of CD-LNMO, LLO, and LNMO with a specific current of 100 mA g^−1^. All the tests are performed in Li-metal coin cells within 2.0–4.8 V at 25 ± 5 °C.

Li||CD-LNMO coin cells were utilized to investigate the electrochemical properties of CD-LNMO. The cells were cycled within 2.0–4.8 V at 25 ± 5 °C. As shown in Fig. 4a, CD-LNMO represents a moderate voltage degradation of ~1.6 mV per cycle, which is comparable to that of LNMO but much smaller than that of LLO (~9.4 mV per cycle). Upon cycling, the discharge capacity of CD-LNMO, LLO, and LNMO increase in the initial cycles (Fig. 4b–e), which may be associated with the gradual activation of electrodes^14^. The reversible capacity of CD-LNMO reached 314.1 mA h g^−1^ (at 9th cycle) under a specific current of 100 mA g^−1^, indicating more removable Li ions than those from LLO (244.5 mA h g^−1^, at specific current of 100 mA g^−1^ for 80 cycles) and LNMO (187.3 mA h g^−1^, at 100 mA g^−1^ for 80 cycles). Taking rocksalt as the reference, partial cation sites in Li_1.46_Ni_0.32_Mn_1.2_O_4–*x*_ were unoccupied due to the release of protons; therefore, more Li ions could be intercalated upon further discharge. The high capacity of CD-LNMO electrode is benefited from the partial cation disorder that enables Li-ion storage in the abundant 16c octahedral sites, and the calculated specific energy of the cathode material in Li||CD-LNMO cell (999.3 Wh kg^−1^ at 9th cycle) is well aligned with the literature research work on insertion-type research cathodes (Supplementary Table 4)^59–61^. In contrast, the increase in spinel-type ordering in the CD-LNMO-H phase gives rise to a lower reversible capacity (243.5 mA h g^−1^, at 100 mA g^−1^ for 50 cycles) and specific energy (766.7 Wh kg^−1^, Supplementary Fig. 16), indicating the crucial role of the entropy state on the electrochemical performance of Li_1.46_Ni_0.32_Mn_1.2_O_4–*x*_. Moreover, CD-LNMO-H suffers from a rapid capacity decay with a specific capacity retention of 74.6% within 50 cycles (initial discharge capacity of 221.8 mA h g^−1^, discharge capacity of 165.1 mA h g^−1^ after 50 cycles, Supplementary Fig. 16), which is much lower than that of CD-LNMO (88.5% within 50 cycles, initial discharge capacity of 250.1 mA h g^−1^, discharge capacity of 221.3 mA h g^−1^ after 50 cycles).

Considering the higher work window of LNMO than that of LLO, LNMO is also cycled within 2.0–5.2 V to provide a comprehensive comparison (Supplementary Fig. 17a, b). The discharge capacity of LNMO reaches 279.3 mA h g^−1^ at a low specific current (30 mA g^−1^), indicating the reversible shuttling of ~1.9 Li^+^ ions per formula. Such a high depth of discharge promotes the phase transition from cubic to tetragonal phases (the discharge plateau located at ~2.0 V) that has been previously revealed to bring higher distortion^15,62^. Therefore, the capacity of LNMO decreasing rapidly with 36.9% (initial discharge capacity of 279.3 mA h g^−1^, discharge capacity of 103.1 mA h g^−1^ after 80 cycles) retained after 80 cycles. For CD-LNMO, the 2.0 V plateau raised by the cubic to tetragonal phase transition in the spinel phase could barely be observed (Fig. 4b). This suppressed phase transition is consistent with the aforementioned XRD/ND results, which facilitates improved cycling stability in CD-LNMO (71.5% capacity retention after 80 cycles at 100 mA g^−1^, initial discharge capacity of 250.1 mA h g^−1^, and discharge capacity of 178.7 mA h g^−1^ after 80 cycles, Fig. 4e) compared to that of conventional spinel (within 2.0–5.2 V at 30 mA g^−1^, 36.9% capacity retention after 80 cycles, initial discharge capacity of 279.3 mA h g^−1^, discharge capacity of 103.1 mA h g^−1^ after 80 cycles; within 2.0–4.8 V at 100 mA g^−1^, 63.9% capacity retention after 80 cycles, initial discharge capacity of 154.1 mA h g^−1^, discharge capacity of 98.5 mA h g^−1^ after 80 cycles, Supplementary Fig. 17b, c) and previously reported high-capacity cathodes (Supplementary Table 4). Upon cycling, the Coulombic efficiency (CE) gradually increased from ~94% in the second cycle to ~99% in the first 30 cycles (at 200 mA g^−1^, Supplementary Fig. 18), which is likely associated with the probable side reactions, e.g., the formation of solid electrolyte interphase, cathode–electrolyte interphase, and the evolution of gas in the initial cycles^59,63^. Operando DEMS was performed to investigate possible side reactions in CD-LNMO (Supplementary Fig. 19), from which we found a noticeable amount of CO_2_ evolution initiated at ~4.5 V. The release of CO_2_ has been consistently revealed in previous work on high-voltage cathodes^59,61^, and a significant portion of the CO_2_ evolution is considered to be raised by side reactions like alkali carbonate decomposition^59,63^. The irreversible release of O_2_, which is one of the most critical issues of Li_*x*_TM_*y*_O_2_, was found to be relatively low in CD-LNMO (~29.1 nmol mg^−1^) compared to that in the previous literature^64^, indicating that only partial O^2−^ is oxidized into O_2_ during the CD-LNMO charging process.

**Fig. 5:**
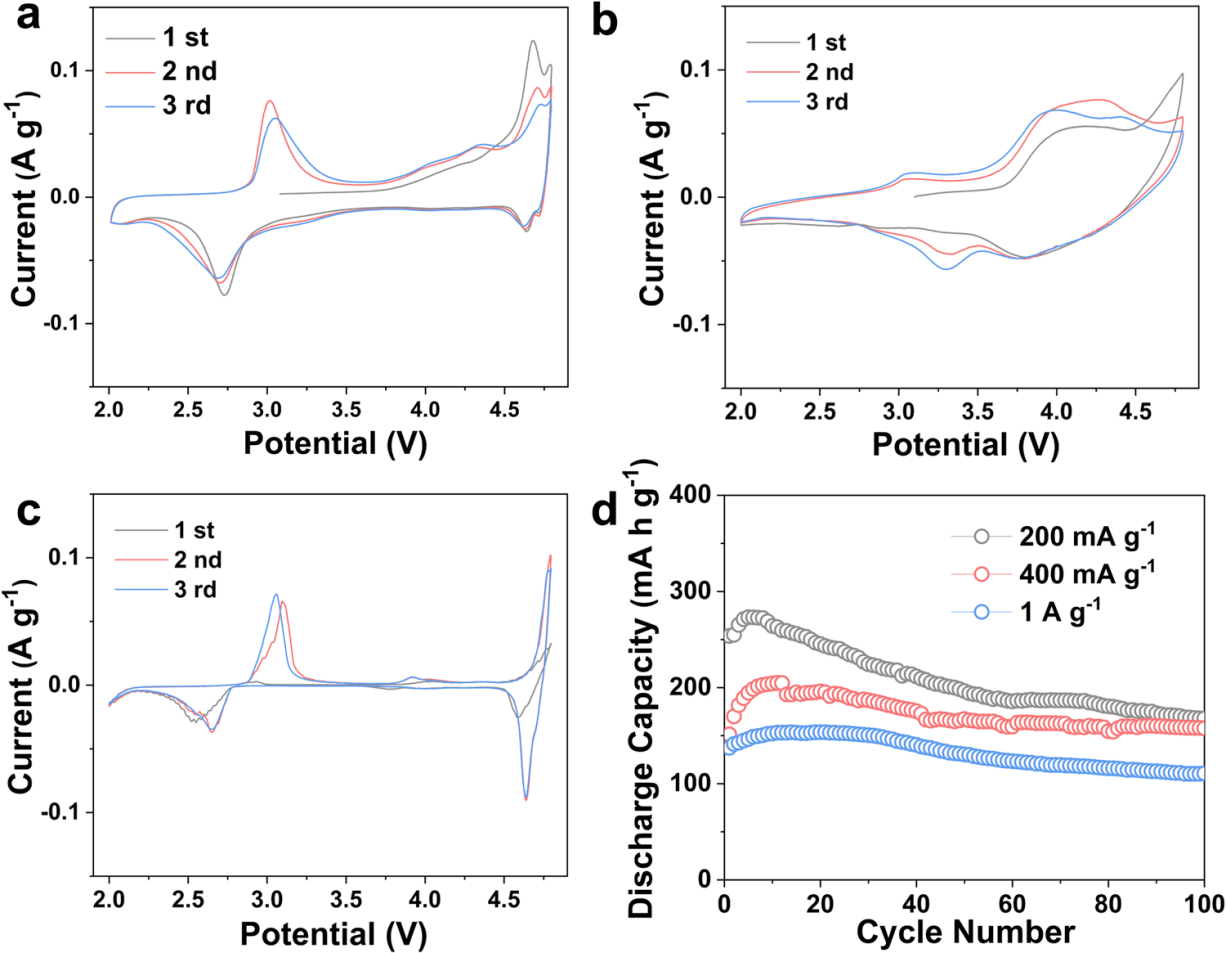
Cyclic voltammetry and cycling stability of various rocksalt-type transition metal oxide materials. **a**–**c** The cyclic voltammograms from the first three cycles of the Li||CD-LNMO (**a**), Li||LLO (**b**), and Li||LNMO (**c**) coin cells. **d** The cycling tests of Li||CD-LNMO cells at various specific currents. CV measurements were performed with a scan rate of 0.05 mV s ^–1^. All the tests are performed in Li-metal coin cells within 2.0–4.8 V at 25 ± 5 °C, therefore the potential value is referred to as the Li/Li^+^ redox couple.

The charge–discharge profiles of CD-LNMO in Fig. 4b show the voltage hysteresis among the first two charging processes, which we speculate is associated with a discrepant redox behavior and a crystallographic change^7,34,35^ after the initial charging process of CD-LNMO. This is consistent with the observation of cation reordering after initial delithiation revealed by XRD and HAADF/STEM. To visualize the shift of the redox center traced from partial cation disorder, cyclic voltammetry (CV) tests were performed and are shown in Fig. 5a–c and Supplementary Fig. 20. The CV curves of CD-LNMO, CD-LNMO-H, and LNMO present two distinct cationic redox peaks centered at ~4.7/4.6 V and ~3.0/2.7 V, corresponding to the Ni^2+^/Ni^4+^ and Mn^3+^/Mn^4+^ redox reactions, respectively. Compared to those of CD-LNMO-H and LNMO, the weak Ni^4+^/Ni^2+^ along with strong Mn^4+^/Mn^3+^ reduction peaks in the CV curve of CD-LNMO are consistent with the short 4.6 V plateau and long 2.7 V plateau in the discharge profile, implying different electrochemical behavior of CD-LNMO. As the energy level of the Ni^4+^/Ni^2+^ redox is lower than that of Mn^4+^/Mn^3+^ in the TMO_6_ configuration^4^, this shifted redox center in CD-LNMO implies incomplete Ni^2+^/Ni^4+^ oxidation within 2.0–4.8 V. It is worth mentioning that the Mn^4+^/Mn^3+^ reduction potential varies with the different localized configurations of Li ions (~4.0 V for LiO_4_ and ~2.7 V for LiO_6_), reflected as the two-step Mn^4+^/Mn^3+^ reduction in LiMn_2_O_4_^47^ and Li_4_Mn_5_O_12_^48^. In contrast, the CV curve of CD-LNMO shows a negligible Mn^4+^/Mn^3+^ reduction peak at 4.0 V, suggesting the electrochemical behavior is different from that of LiMn_2_O_4_ and Li_4_Mn_5_O_12_. In comparison, both the CD-LNMO-H and LNMO electrodes present more of the Ni^4+^/Ni^3+^ reduction reaction than CD-LNMO (Supplementary Fig. 20b), which represents the altered redox reaction after entropy manipulation. Moreover, the scan rate-dependent CV measurements (Supplementary Fig. 21) revealed diffusion-controlled faradaic behavior in the charging process and the coexistence of non-faradaic and faradaic behavior upon discharging, suggesting at least a partial surface redox reaction in the low voltage region^65^. Benefiting from the rapid Li^+^ diffusion along the 0-TM path under TM-poor conditions^66^ and in the spinel framework, Li||CD-LNMO coin cells show promising rate performance with reversible capacities of 273.1 mA h g^−1^, 204.9 mA h g^−1^ and 153.6 mA h g^−1^ under increased specific currents (200 mA g^−1^, 400 mA g^−1^, and 1 A g^−1^), which results in calculated specific energy values of 803.9, 634.6 and 420.9 Wh kg^−1^ for the cathode active material in coin cells, respectively (Fig. 5d). The rate performances of LLO and LNMO (Supplementary Fig. 22) were substantially restricted by the sluggish reaction kinetics of the positive electrodes^34,45^, and the calculated specific energy values of the cathode materials in Li-metal coin cell were 303.9 and 154.2 Wh kg^−1^ (at a specific current of 1 A g^−1^), respectively.

### Ex situ physicochemical characterizations and atomistic calculations of Li_1.46_Ni_0.32_Mn_1.2_O_4–*x*_-based electrodes

**Fig. 6:**
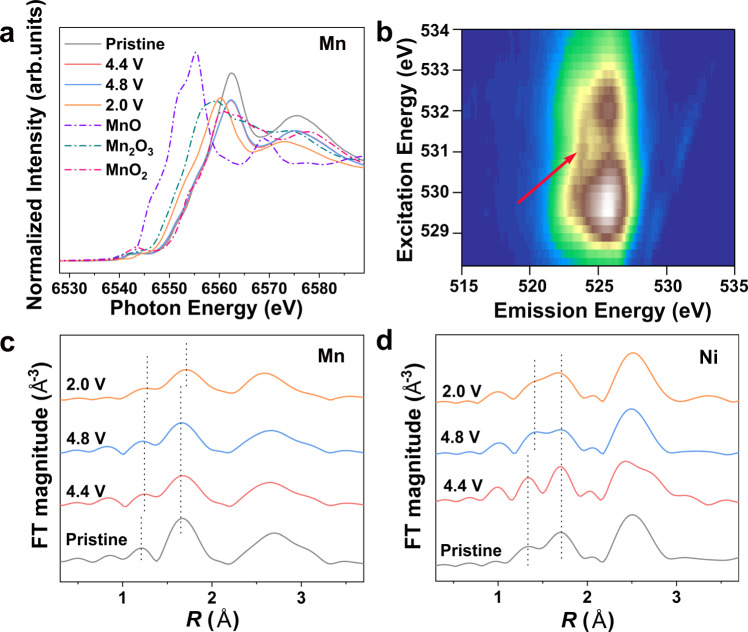
Ex situ X-ray characterizations of Li_1.46_Ni_0.32_Mn_1.2_O_4–*x*_-based electrodes. **a** Normalized ex situ Mn K-edge XANES spectra obtained from CD-LNMO electrodes at different voltages during the first cycle. The Mn K-edge XANES spectra of MnO, Mn_2_O_3_, and MnO_2_ are shown for reference. The 4.4 V and 4.8 V curves are charged states, and the 2.0 V curve is the discharge state. The cells are cycled within 2.0–4.8 V at a specific current of 100 mA g^−1^ under 25 ± 5 °C. **b** O-K-edge mRIXS mapping of 4.8 V charged electrodes obtained with the specific current of 100 mA g^−1^. The oxidized oxygen feature is marked by a red arrow. **c**, **d** ***k***^3^-weighted Fourier transform magnitudes of **c** Mn K-edge EXAFS spectra and **d** Ni K-edge EXAFS spectra obtained from CD-LNMO electrodes at different voltages during the first cycle. The position of the first two shell coordination peaks (within 1 ~2 Å) is marked by dashed lines.

Ex situ Mn and Ni K-edge X-ray absorption near-edge structure (XANES) measurements were then adopted to probe the oxidation state change of Mn and Ni in CD-LNMO during the initial cycle. The absorption edges of Mn and Ni in the as-synthesized CD-LNMO are close to those of MnO_2_ and NiO, indicating the near +4 and +2 valence states for Mn and Ni, respectively (Fig. 6a and Supplementary Fig. 23). During the initial cycle, the valence state of Mn barely changed during the charging process but approached +3 in the fully lithiated state (2.0 V). Moreover, the slight shift of the absorption edges in the Ni K-edge XANES spectra revealed the partially Ni^2+^/Ni^3+^ redox reaction (Supplementary Fig. 23). Thus, it is likely that the initial charging process is mostly charge compensated by anionic oxidation, while the accompanying structural evolution triggers Mn^4+^/Mn^3+^ redox during the discharge process. The high-efficiency mapping of resonant inelastic X-ray scattering (mRIXS), which enables the direct observation of oxidized oxygen^67,68^, was therefore conducted on the fully charged electrode to detect the reversible anion redox. As shown in Fig. 6b and Supplementary Fig. 24, the mRIXS of the 4.8 V charged sample represents a strong O^*n*-^ (*n* < 2) feature at ~531 eV excitation and ~524 eV emission (marked by red arrow), suggesting considerable oxidized oxygen in the lattice of fully delithiated CD-LNMO. Combining with the DEMS and ex situ O 1 *s* XPS results (Supplementary Fig. 25), we can confirm the presence of a reversible anion redox in the initial cycle of CD-LNMO. The change in TM-O bonding in the initial cycle was also characterized by ex situ O K-edge soft X-ray absorption spectroscopy (sXAS) measurements. The energy of the ligand K-edge pre-edge reflects the energy level of the lowest unoccupied molecular orbital (LUMO), which is dominated by the *t*_2g_ and *e*_g_ orbitals of Mn/Ni^64^. Therefore, the increased intensity of pre-edge peaks upon charging indicates strengthened TM-O hybridization, while the slight shift in the first pre-edge peak in the fully lithiated sample (2.0 V) reflects the reduction of Mn/Ni ions (Supplementary Fig. 26)^69^.

Turning to the localized structure, the ex situ Mn and Ni K-edge EXAFS revealed the TM-O bond evolution within the initial cycle (Fig. 6c, d). Upon charging, the shorter MnO bond (<1.5 Å) was slightly stretched, while the longer MnO bond (>1.5 Å) remained unchanged (Fig. 6c). This is likely to be raised by the oxidation of O ions and the rearrangement of Mn ions that diminish the localized distortion of MnO_6_ ligands. The MnO bonds were further stretched during the discharge process, most likely caused by the reduction of Mn ions. For the Ni-O bond, as shown in Fig. 6d, the relative intensity of the peak corresponding to the shorter Ni-O bond (<1.5 Å) increased during the charging process and decreased upon discharge, which seems to be associated with both Ni reordering and the Jahn-Teller effect of Ni^3+^ under 4.8 V. The stretching of the shorter Ni-O bond implies the diminished localized distortion of the NiO_6_ ligand in the charging process, while the slight Ni^3+^/Ni^2+^ reduction reaction upon discharge barely influenced the Ni-O bond length. Moreover, the ex situ SXRD measurements revealed a reserved spinel-type long-term ordering after the 80th cycle (Supplementary Fig. 27), confirming diminished spinel to rocksalt structure degradation upon cycling.

**Fig. 7:**
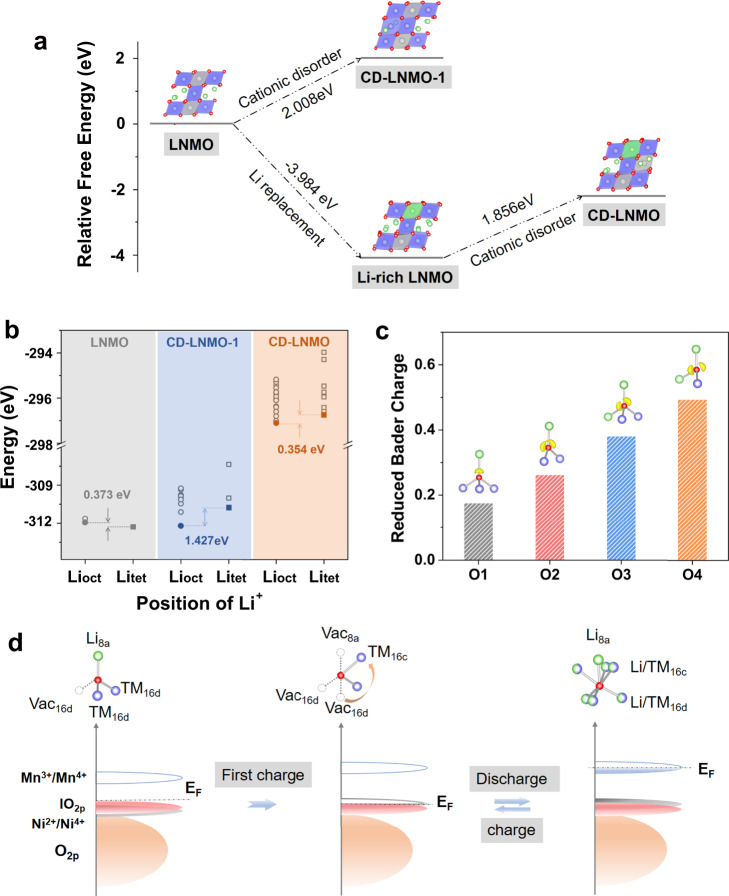
Understanding the physicochemical properties of lithium-rich layered oxide materials via atomistic calculations. **a** Calculated free energy diagrams of the structural evolution on LNMO. The ligands LiO_6_, MnO_6_, and NiO_6_ are represented by green, blue, and gray octahedrons, respectively. The free energy values were calculated based on the total supercell. **b** The energy difference between the octahedral sites (Li_oct_) and tetrahedral sites (Li_tet_) in LNMO, CD-LNMO-1, and CD-LNMO. **c** The reduced Bader charges of oxygen ions under different conditions of the fully delithiated CD-LNMO. The insets show the calculated ELF (isovalues of 0.7) of the oxygen ions in each condition. The Li, TM, and O ions are represented by green, blue and red balls, respectively. **d** Schematic illustration of the localized conditions and corresponding electronic structure evolution upon cycling. The Li, TM, and O ions are shown as green, purple, and red balls, respectively, and the vacancies are represented as hollow dash cycles. The redox of Mn^3+^/Mn^4+^ and Ni^2+^/Ni^4+^ are expressed by blue and gray bands, respectively, and lone pair O 2*p* orbitals and paired O 2*p* orbitals are marked by lO_2*p*_ (red bands) and O_2*p*_ (orange bands), respectively. The Fermi level, *E*_F_, is shown as a dashed line.

To elucidate the correlation between cation disorder and the electrochemical properties of CD-LNMO, we performed DFT calculations to investigate the crystal and electronic structures of CD-LNMO. Herein, both cation disordering and Li over-stoichiometry were considered in the LiNi_0.5_Mn_1.5_O_4_ structure (space group: *Fdm*), which helps to investigate the localized crystal and electronic structural changes in CD-LNMO. The modeling and calculation steps are illustrated in Supplementary Note 3 and Supplementary Fig. 28. As shown in Fig. 7a, we found that cation mixing in stoichiometric LNMO, marked as CD-LNMO-1, is thermodynamically unfavorable (formation energy (*E*_*x*_) of 2.008 eV). In contrast, the formation of CD-LNMO, whose fully lithiated state was proven in the above experiment to be a Li-rich spinel with cation disordering, is more favorable than that of CD-LNMO-1. This is attributed to the substitution of TM ions by an excess of Li ions, which reduces the Coulombic repulsion between nearby octahedral sites and thermodynamically facilitates cation disordering in Li-rich LNMO. The influence of cation disordering on the Li-ion storage mechanism was then explored by comparing the site energies of the 16c octahedral sites (Li_oct_) and 8a tetrahedral sites (Li_tet_). As shown in Fig. 7b, the site energy of Li_tet_ is 0.373 eV, lower than that of Li_oct_ in LNMO, which is consistent with previous understanding^18^ and implies a preferential insertion of Li ions into the tetrahedral sites. In contrast, the lowest site energies of Li_tet_ are 1.431 eV and 0.354 eV higher than those of Li_oct_ in CD-LNMO-1 and CD-LNMO, respectively, indicating the prioritized occupation of octahedral sites. Such variation in the site energy can be traced from TM ion migration from the 16d to 16c sites. Specifically, the occupancy of 16c interstitial sites results in thermodynamically unfavorable face sharing of the TMO_6_ ligand with the adjacent LiO_4_ ligand, which raises the site energies of Li_tet_, while the created 16d vacancy reduces the site energies of the nearby Li_oct_. This site energy variation prevents the insertion of Li ions into the Li_tet_ sites, consistent with the shortened 4.7 V plateau and prolonged 2.7 V plateau in the previous paragraphs.

Cation disordering and an excess of Li inevitably alter the localized condition of oxygen ions. For LNMO, all of the oxygen ions are coordinated by 3TM1Li, whereas new localized oxygen conditions coordinated by 2TM1Li (oxygen ions near the 16d vacancy), 2TM2Li (oxygen ions near the excess Li in 16d sites), and 1TM2Li (oxygen ions between the vacancy and excess Li ions) are generated in CD-LNMO. These were labeled as O1 (3TM1Li), O2 (2TM1Li), O3 (2TM2Li), and O4 (1TM2Li), respectively. As shown in the inset of Fig. 7c, the calculated electron localization function shows the O-2*p* lone pair orbitals (lO_2*p*_) on O2, O3, and O4, implying a higher energy level of the anionic reaction on these oxygen ions. This was further proven by the reduced Bader charge (per atom) upon full delithiation of CD-LNMO (Fig. 7c), in which the oxygen ions coordinated with fewer TM ions, representing a higher degree of oxidation. Moreover, no O-O dimer was formed in the fully delithiated CD-LNMO (Supplementary Fig. 29), suggesting that the anionic reaction is insufficient to trigger oxygen release and thus contributes a reversible capacity.

In summary, the ex situ X-ray measurements support the claim of the change in the redox center and the migration of Mn ions in the initial cycle, and the DFT calculations revealed the altered Li storage mechanism and anionic redox in the medium-entropy state phase. These results are in line with the aforementioned STEM and electrochemical observations and therefore help us build an outline of the crystal and electronic structures of CD-LNMO upon cycling. As shown in Fig. 7d, anionic oxidation dominates charge compensation in the first charge, and the observation of lO_2*p*_ in CD-LNMO reveals a higher energy level of O^2−^/O^−^ than that of Ni^2+^/Ni^4+^, suggesting at least partial O^2−^ ions located in a TM-poor spinel structure. Upon removal of the Li ions, the migration of TM ions and anionic oxidation trigger irreversible structural changes that lower the energy level of the O^2−^ ions, therefore enabling reversible Mn^4+^/Mn^3+^ redox accompanied by Li ions inserted/extracted mostly from the octahedral sites. This endows reversible cycling through both cationic and anionic redox, and the shuttling of Li ions through octahedral sites not only gives rise to the boosted capacity but also effectively avoids symmetry changes upon high-capacity operation.

## Discussion

Regarding the wide gap between the practical and theoretical capacities of Li_*x*_TM_*y*_O_2_ cathodes, the intrinsic symmetry change of Li_*x*_TM_*y*_O_2_ upon high-capacity operation is one of the most crucial challenges to be overcome. Herein, we demonstrated that a suitable entropy level could effectively suppress the undesired symmetry change, enabling high-capacity operation through a 3D Li-ion diffusion path. We found that a medium-entropy state generated through partial cation disordering alters the Li-ion storage mechanism and circumvents the symmetry change in the conventional spinel phase. The ex situ SXRD and ND measurements revealed shuttling of Li ions from octahedral sites and the diminished spinel to T1/T2 phase transitions upon cycling, while the XPS, XAS, DEMS, and DFT calculations coherently revealed the oxidation of O^2−^ in the initial cycle and the Mn^4+^/Mn^3+^ reduction reaction during discharge. These findings indicate that the entropy state would effectively tune the crystallographic and electronic structures of cathode materials; therefore, an appropriate entropy level is crucial to reach an optimized electrochemical performance in each system. Looking forward, the entropy manipulation strategy presented in this work is a potential direction to overcome the thermodynamically driven atomic evolution upon the insertion/extraction of Li ions, and the cation-disordered spinel structure opens up a large space for exploring medium-entropy compounds.

## Methods

### Materials synthesis

Layered-type Li_1.2_Ni_0.2_Mn_0.6_O_2_ was synthesized through a co-precipitation method with sodium sulfide nonahydrate (Na_2_S·9H_2_O, 99.99%) and sodium carbonate (Na_2_CO_3_, 99.5%) as precipitating agents. Stoichiometric manganese sulfate monohydrate (MnSO_4_·H_2_O, 98%, 6 mmol) and nickel sulfate hexahydrate (NiSO_4_·6H_2_O, 98%, 2 mmol) was dissolved into 10 mL deionized water as the source of transition metal ions, and the precipitating solution was prepared by dissolving 1 g Na_2_CO_3_ and 1 g Na_2_S·9H_2_O into the mixture of 25 mL deionized water and 15 mL ethanol. After 1 h stirring at 25 ± 5 °C, the precipitating solution was dropwise added into the mixture of MnSO_4_·H_2_O and NiSO_4_·6H_2_O under continuous stirring, and the obtained precursors were mixed with 10 at% excessive lithium hydroxide monohydrate (LiOH·H_2_O, 98%) before calcinated at 800 °C in the air for 12 h with a ramping rate of 3.5 °C /min. The obtained products were washed with deionized water and ethanol five times to remove the unreacted residuals. The entropy of Li_1.46_Ni_0.32_Mn_1.2_O_4–*x*_ was manipulated through a two-step treatment of Li_1.2_Ni_0.2_Mn_0.6_O_2_. A proton exchange process was performed through 90 °C acid treatment (0.1 M HNO_3_, diluted by 70% HNO_3_ purchased from Sigma-Aldrich Reagent) of Li_1.2_Ni_0.2_Mn_0.6_O_2_ for 1 h, the products were washed by deionized water and ethanol several times to remove the adsorbed acids. The highly defective Li_1.46_Ni_0.32_Mn_1.2_O_4–*x*_ phase was prepared by low-temperature calcination (500 °C) in the air for 1 h. Li_1.46_Ni_0.32_Mn_1.2_O_4–*x*_ phase with more thorough cation reordering was synthesized as a comparison, which is obtained by higher temperature treatment (700 °C) in the air for 1 h. All of these chemicals were purchased from Sigma-Aldrich Reagent (analytical reagent) and used without any further purification, while the deionized water (~17.5 MΩ cm) is produced by IQ 70XX (Milli-Q) and the ethanol is purchased from Sinopharm Chemical Reagent (95%, analytical reagent) without any further purification. Commercial LiNi_0.5_Mn_1.5_O_4_ (>99% without carbon coating, average particle size of ~10 μm) was purchased from Guangdong Canrd New Energy Technology Co., Ltd.

### Ex situ physicochemical characterizations

The morphology of products was observed by TEM (FEI Tecnai G^2^ F20), while the phase constitution was detected by XRD (PANalytical–Empyrean) equipped with Cu Kα radiator (40 kV, 40 mA). ICP–MS measurement (Agilent 7700) was performed to quantify the chemical composition of obtained products. The samples for ICP–MS test are dissolved in aqua regia before measurements. The thermogravimetric analysis (TGA) was carried out on a TG instrument (Q50 TGA) with a Pt crucible, and the test is carried out in air with a ramping rate of 5 °C min^–1^.

### X-ray spectroscopy

All the XPS measurements were carried out on a Thermo Fisher Scientific VG Kα Probe spectrometer, and the obtained spectra were calibrated by the C 1 *s* peak (284.8 eV). Each ex situ XPS measurement was performed after 30 s Ar etching under 500 V to remove the surface residuals, and the etching depth is estimated to be ~3 nm based on previous reports^70^. The XPS fittings were conducted with a fixed Lorentzian/Gaussian (L/G) ratio of 20% and a restricted full width at half maximum within 1.2~1.6. The K-edge XANES and EXAFS spectra of Mn and Ni were performed on the 4B9A beamline in Beijing Synchrotron Radiation Facility (BSRF). The storage rings of BSRF were operated at 2.5 GeV with a stable current of 400 mA. Using Si (111) double-crystal monochromator, the data collection was carried out in fluorescence mode using Lytle detector. Soft XAS measurements were carried out on the Beamlines MCD-A and MCD-B (Soochow Beamline for Energy Materials) at NSRL. All the XAS data were processed and analyzed by the Athena program, and the Mn and Ni EXAFS spectra are obtained with the *k* range from 2.6–12 and 2.6–13.5, respectively. The *k* weight is 3. Samples for ex situ XPS, XAS and RIXS measurements were charged/discharged to given voltages in Li-metal coin cells and dissembled immediately inside an Ar-filled glovebox (H_2_O, O_2_ < 0.5 ppm), and the obtained electrodes are directly utilized without scraped from current collector. The electrodes were immersed in dimethyl carbonate (10 mL, 99%, Sigma-Aldrich Reagent) for 24 h, followed by five times rinsing by dimethyl carbonate to remove surface residuals. For all the ex situ XPS and SXAS measurements, the electrodes were placed on sample holders and sealed in Ar-filled homemade protectors, the samples were stored inside an Ar-filled glovebox (H_2_O, O_2_ < 0.5 ppm) before being transferred into the detection chamber. Additionally, the samples for ex situ Mn and Ni K-edge XAS measurements are sealed by Kapton tape (8 μm, SPEX) throughout the tests to avoid any possible oxidation. O K-edge RIXS mapping was performed in the iRIXS end station. The electrode was placed on a copper sample holder (height of 1.27 cm, and diameter of 2.54 cm) before being transferred into the ultra-high vacuum chamber (10^−10^ Torr), and the whole process was conducted in an Ar atmosphere to avoid potential air exposure. The excitation energy resolution is ~0.2 eV. The excitation photon energy was calibrated by the first peak of the TiO_2_ reference sample as 531 eV, while the emission energy was calibrated by the elastic peak. The RIXS spectra were collected for 90 s at each excitation energy.

### Synchrotron X-ray and neutron diffraction

The SXRD tests were performed on the BSRF, which used dual-focus monochrome X-rays provided by the 1W1A beamline. The wavelength of the X-ray was 1.54 Å. The time-of-flight ND measurements of the charged and discharged Li_1.46_Ni_0.32_Mn_1.2_O_4–*x*_ were carried out on Multiple-Physics Instrument beamline at the China Spallation Neutron Source. The ND patterns were collected at 298 K. For the ex situ SXRD and ND measurements, the charged and discharged Li_1.46_Ni_0.32_Mn_1.2_O_4–*x*_ were prepared by assembling Li-metal pouch cells with the total active material of ~500 mg. The pouch cells were cycled under 20 mA g^−1^ to ensure thorough delithiation and lithiation, and dissembled immediately inside an Ar-filled glovebox (H_2_O, O_2_ < 0.5 ppm) at given voltages. The electrodes were immersed in dimethyl carbonate (50 mL, 99%, Sigma-Aldrich Reagent) for 24 h, followed by five times rinsing by dimethyl carbonate to remove surface residuals. The samples were scraped from Al foil for the following tests. For SXRD measurements, the samples are sealed by Kapton tape (8 μm, SPEX) inside an Ar-filled glovebox (H_2_O, O_2_ < 0.5 ppm), and the sealed samples are directly transferred onto the sample holder and detection chamber in air. For ND measurements, the samples are placed onto the sample holder before being transferred into the detection chamber, and the whole process was conducted in an Ar atmosphere to avoid potential air exposure. As the neutron flux is much lower than that of X-ray, most of the active materials (~80%) were sealed inside a vanadium can for the ND measurements, while the remaining (~20%) were sealed by Kapton tape for SXRD tests. Both the SXRD and ND refinements were refined by GSAS-II software, and the refinements are performed sequentially (first SXRD, then ND). During the SXRD refinement, we performed a two-step refinement that refined and subtracted the background from Kapton tape and the fluorescence effect of Ni and Mn before the final refinement; the lattice constants, atomic occupancies of TM ions, instrument parameters, and peak broadening were refined, while the obtained parameters (lattice constants, and atomic occupancies of TM ions) were utilized in the following ND refinements. The ND refinements were carried out to analysis the occupancies of Li ions, therefore the lattice constants, atomic occupancies of Li ions, instrument parameters, and peak broadening were refined with fixed atomic positions and occupancies of TM ions.

### Differential electrochemical mass spectrometer measurements

The gas release was detected on a Differential Electrochemical Mass Spectrometer (i-DEMS 100). The specific Swagelok-type cylindrical cell with an inner diameter of 22 mm was assembled in an Ar-filled glovebox (H_2_O < 0.01 ppm, O_2_ < 0.01 ppm), with the CD-LNMO electrode (75 wt% of active materials, 1 × 1 cm^2^ square, ~1 mg, thickness of ~10 μm) and a Li foil (>99%, Φ16 × 0.5 mm, China Energy Lithium) as the working electrode and counter/reference electrode, respectively. The cell was filled by 100 μL 1 M LiPF_6_ in ethylene carbonate (EC)/dimethyl carbonate (DMC) (H_2_O < 20 ppm, LBC502A50, CAPCHEM), and the measurement utilized Argon (>99.999%) as carrier gas with the flow rate of 0.9 ml min^−1^. Electrochemical measurements were performed on a Landt battery tester (LANHE, Wuhan) with a specific current of 60 mA g^−1^ (based on the weight of the positive electrode’s active material). At the same time, the released O_2_ and CO_2_ were collected and quantified by the mass spectrometer.

### HAADF-STEM and EELS characterization

A double aberration-corrected STEM (FEI Titan Cubed G^2^ 60–300) operated at 300 kV was explored to perform the HAADF and EELS measurements. The contrast of the HAADF image is determined by the average atomic number of the atomic columns (~*Z*^1.5^ to *Z*^1.8^). The pristine sample for STEM measurement was ground and sonicated in an ethanol solution, and the supernatant was collected by the carbon grid. To prepare the fully charged sample for STEM tests, the Li-metal coin cell was dissembled immediately after charged to 4.8 V in the initial cycle, followed by rinsing with the same procedure of ex situ XPS/XAS samples. The active materials were scraped from the Al foil and ground in anhydrous hexane (50 mL, 95%, Sigma-Aldrich Reagent) for 30 min before being deposited onto the carbon grid (carbon membrane purity of >90%, thickness of 3–5 nm, 200 mesh, Electron Microscopy China). All the procedures are carried out inside an Ar-filled glovebox (H_2_O, O_2_ < 0.5 ppm). To avoid possible oxidation, the carbon grid was sealed into an Ar-filled container and stored in Ar-filled glovebox (H_2_O, O_2_ < 0.5 ppm) before being transferred into the sample holder and the STEM column.

### Electrochemical measurements

The positive electrodes of Li-metal cells were prepared by mixing 75 wt% active materials, 15 wt% conductive carbon additive (>90%, average particle size of ~40 nm, MTI), and 10 wt% poly (vinylidene fluoride) (PVDF, Alfa Aesar) in the *N*-methyl-2 pyrrolidone (99.5%, Alfa Aesar) solution. The obtained slurry was spread onto the current collector (Al foil, >99%, 0.1 mm, Times Aluminum Foil) and dried at 60 °C overnight. Before being transferred into the Ar-filled glovebox (H_2_O, O_2_ < 0.5 ppm), the electrodes were cut into 0.5 × 0.5 cm^2^ squares with the loading of ~1 mg cm^−2^ (thickness of ~10 μm) and dried at 120 °C for 10 h in a vacuum oven. The Li-metal cells (coin cell, CR2025) were assembled inside an Ar-filled glovebox (H_2_O, O_2_ < 0.5 ppm), Li foil (>99%, diameter 16 mm and thickness 500 µm, China Energy Lithium), Celgard 2400 membrane (25 μm, average pore size of 0.043 μm, porosity of 41%) and 0.18 mL 1 M LiPF_6_ in EC/DMC (H_2_O < 20 ppm, LBC502A50, CAPCHEM) were utilized as the anode, separator, and electrolyte, respectively. The galvanostatic charge/discharge tests were performed on CT2001A battery test systems (LAND Wuhan Corp., China), and the CV data were collected on an electrochemical workstation (CHI 660 C) (Shanghai Chenhua Instrument Corp., China) with the scan rate of 0.05 mV s^−1^. The scan rate depended CV was performed with the scan rates of 0.2, 0.4, 0.5, 0.8, and 1.0 mV s^−1^ within 2.0–4.8 V. All the electrochemical tests are performed in the room without climatic/environmental chamber, while the temperature is 25 ± 5 °C (controlled by central air-conditioning). The specific energies of cathode materials were calculated based on the integral area on the discharge profile of Li-metal cells, and the weight was based on the positive electrode’s active material to make a parallel comparison with that in previous works (Supplementary Table 4). The pouch cells for ex situ SXRD and ND measurements are assembled within the ~6 × 12 cm^2^ Al bags. The electrodes were single-side coated, and were cut into ~4 × 8 cm^2^ squares with the loading of ~15 mg cm^−2^ (thickness of ~80 μm) before drying at 120 °C for 10 h in a vacuum oven. The Li-metal pouch cells were assembled inside an Ar-filled glovebox (H_2_O, O_2_ < 0.5 ppm), and each cell employs one single-side coated electrode. Li foil (>99%, ~5 × 9 cm^2^, 0.2 mm, China Energy Lithium), Celgard 2400 membrane and 0.64 mL 1 M LiPF_6_ in ECDMC) (H_2_O < 20 ppm, LBC502A50, CAPCHEM) were utilized as the anode, separator, and electrolyte, respectively. The pouch cells were cycled under 20 mA g^−1^ at 25 ± 5 °C to ensure thorough delithiation and lithiation.

### DFT calculations

The structure construction with various configurations was illustrated in the Supplementary Information (Supplementary Fig. 28). The energy of structures and the information of electronic structure were obtained by DFT calculations, which were performed in the Vienna *ab* initio simulation package^71^. The projector augmented wave (PAW) framework was used to describe the core electrons^72^. The exchange-correlation energy was evaluated using the generalized gradient approximation (GGA) with the Perdew-Burke-Ernzerhof functional^73^. The rotationally averaged Hubbard U correction was used to correct the self-interaction error in GGA calculations. Effective U values of 6.0 and 3.9 eV were chosen for the d electrons of Ni and Mn atoms, as these have been reported to produce reasonable results^74,75^. The energy cutoff of the plane wave basis set was set at 520 eV. Gamma-centered *k*-points meshes with a density of 1000 divided by the number of atoms were used for Brillouin-zone integration. Each structural optimization fully relaxed the cell parameters and atomic positions until the force per atom in the supercell was <0.01 eV/Å.

### Reporting summary

Further information on research design is available in the Nature Research Reporting Summary linked to this article.

## Supplementary information

Supporting information

Reporting Summary

## Data Availability

The data generated in this study are provided in Source data. Extra data that support the findings of this study are available from the corresponding authors upon reasonable request. Source data are provided with this paper.

## Source data

Source Data
