# Supplementary material for: A medium-entropy transition metal oxide cathode for high-capacity lithium metal batteries
Source: Nat Commun. 2022 Oct 18;13:6158. doi: 10.1038/s41467-022-33927-0 (PMC9579144; doi:10.1038/s41467-022-33927-0)
Supplement: Supplementary file 1 — Supporting information [file 41467_2022_33927_MOESM1_ESM.docx]

*Supplementary Information for*

**A medium-entropy transition metal oxide cathode for high-capacity lithium metal batteries**

Yi Pei ^1, 9^, Qing Chen ^1, 9^, Meiyu Wang ^2^, Pengjun Zhang ^3^, Qingyong Ren ^4,5^, Jingkai Qin ^1^, Penghao Xiao ^6^, Li Song ^3^, Yu Chen ^4^, Wen Yin ^4, 5^, Xin Tong ^4, 5^, Liang Zhen ^1, 7*^, Peng Wang ^2, 8*^ & Cheng-Yan Xu ^1, 7*^

^1^ Sauvage Laboratory for Smart Materials, School of Materials Science and Engineering, Harbin Institute of Technology (Shenzhen), Shenzhen 518055, China

^2^ National Laboratory of Solid State Microstructures, College of Engineering and Applied Sciences, Collaborative Innovation Center of Advanced Microstructures and Center for the Microstructures of Quantum Materials, Nanjing University, Nanjing 210093, China

^3^ National Synchrotron Radiation Laboratory, CAS Center for Excellence in Nanoscience, University of Science and Technology of China, Hefei 230029, China

^4^ Institute of High Energy Physics, Chinese Academy of Sciences, Beijing 100049, China

^5^ Spallation Neutron Source Science Center, Dongguan 523803, China

^6^ [Department of Physics & Atmospheric Science](https://www.dal.ca/faculty/science/physics.html), Dalhousie University, Halifax, NS B3H 4R2, Canada

^7^ School of Materials Science and Engineering, and MOE Key Laboratory of Micro-Systems and Micro-Structures Manufacturing, Harbin Institute of Technology, Harbin 150001, China

^8^ Department of Physics, University of Warwick, Coventry CV4 7AL, UK

^9^ These authors contributed equally: Yi Pei, Qing Chen

E-mail: wangpeng@nju.edu.cn; lzhen@hit.edu.cn; cy_xu@hit.edu.cn


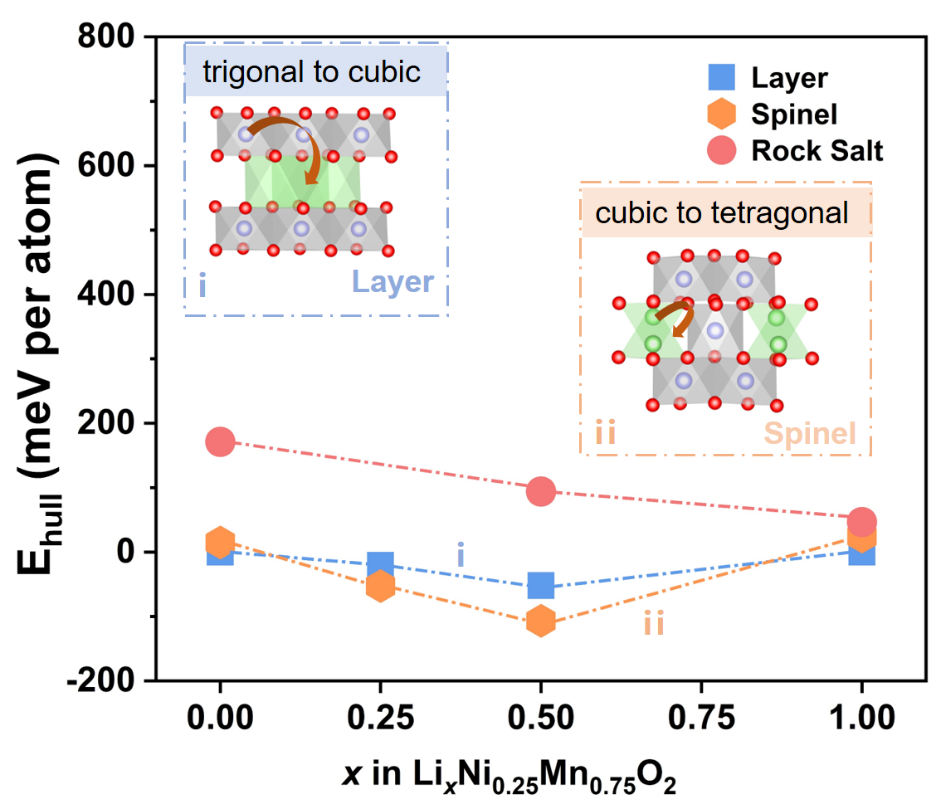


**Supplementary Figure 1 | Energy above the convex hull (*E*_hull_) of the various polytypes of Li*_x_*Ni_0.25_Mn_0.75_O_2_ (0<*x*<1).** The TMO_6_ and LiO_6_ octahedral sites are represented by gray and green octahedrons, respectively; while the LiO_4_ tetrahedral sites are shown as green tetrahedrons. The transition metal ions, Li ions, and oxygen ions are shown as purple, green, and red balls. Insets show the two probable structural evolutions upon de/lithiation.


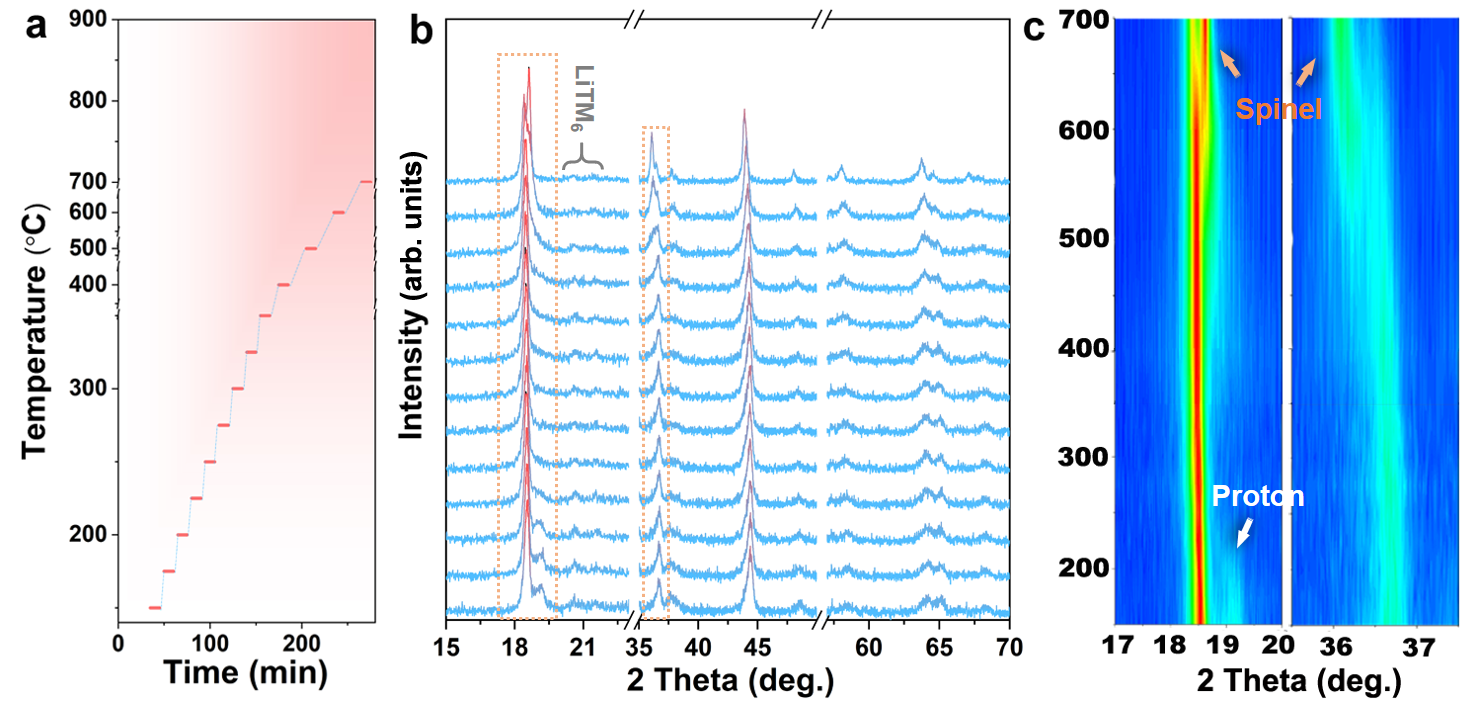


**Supplementary Figure 2 | In situ high-temperature XRD pattern. a.** The calcination process of XRD measurements. **b.** Overall *in-situ* XRD patterns from 150–700 °C. **c.** Highlighted regions within 17–20° and 35.5–37.5°. The reflections of LiTM_6_ superstructure (2 theta within 20–22°) are marked in b, while reflections raised from proton exchanged region and 3D spinel-type ordering are marked by white and orange arrows in **c**, respectively.


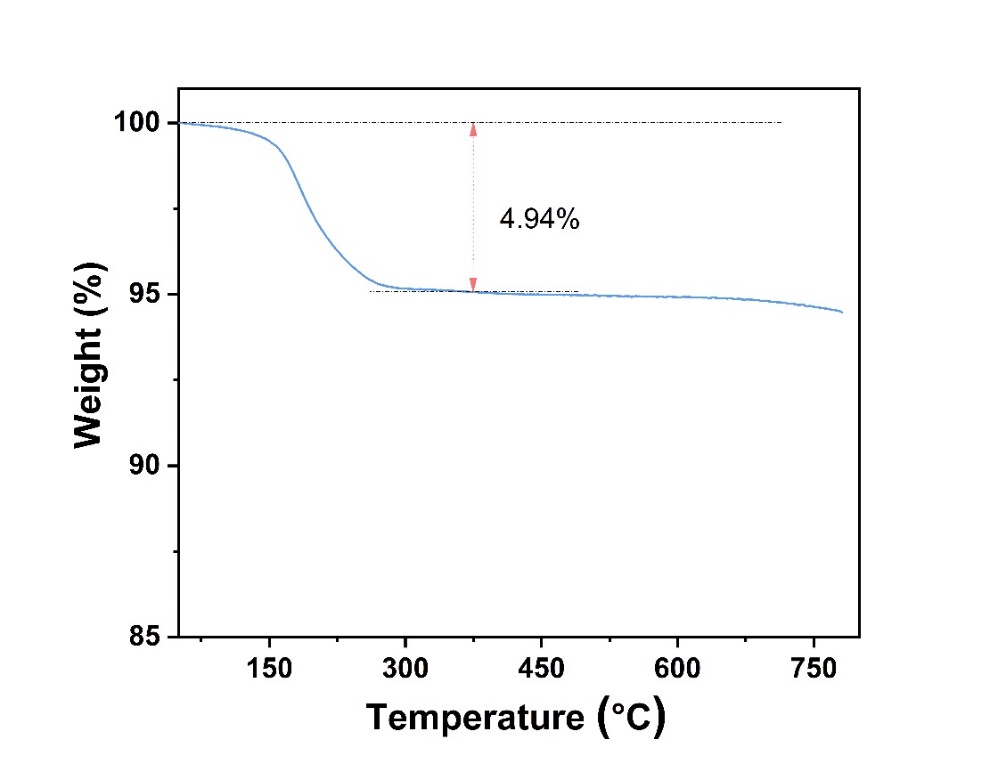


**Supplementary Figure 3 | Thermogravimetric curve of the precursor after proton exchange.** The test is carried out in the air with a ramping rate of 5 °C min^–1^.


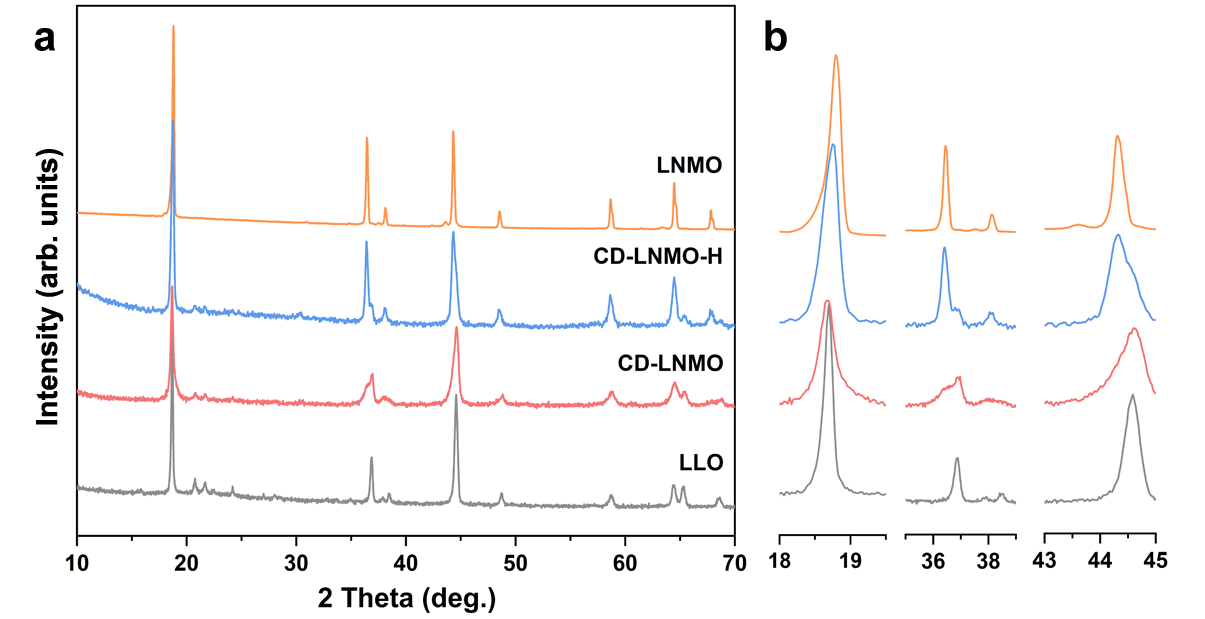


**Supplementary Figure 4 | XRD patterns of as-synthesized samples. a.** Overall XRD patterns of LLO, CD-LNMO, CD-LNMO-H and LNMO. **b.** The amplified region within the 2 theta degree of 18–19.5°, 35–39° and 43–45°.


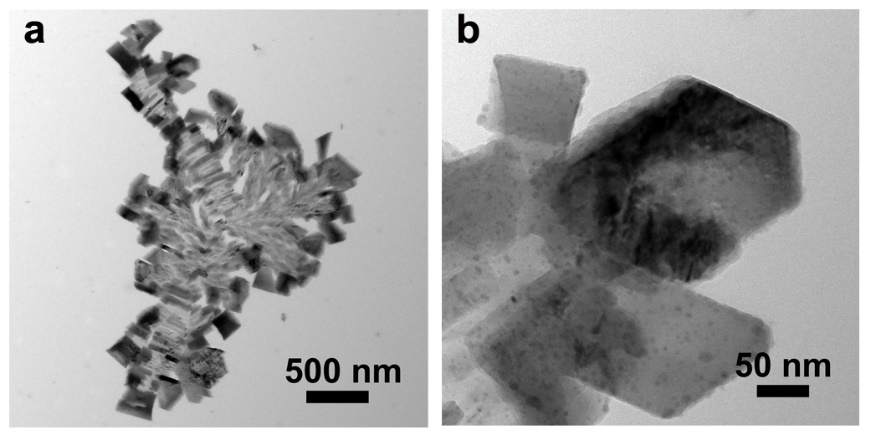


**Supplementary Figure 5 | Morphology of as-synthesized CD-LNMO. a.** Low-magnification TEM image. **b.** High-magnification TEM image.


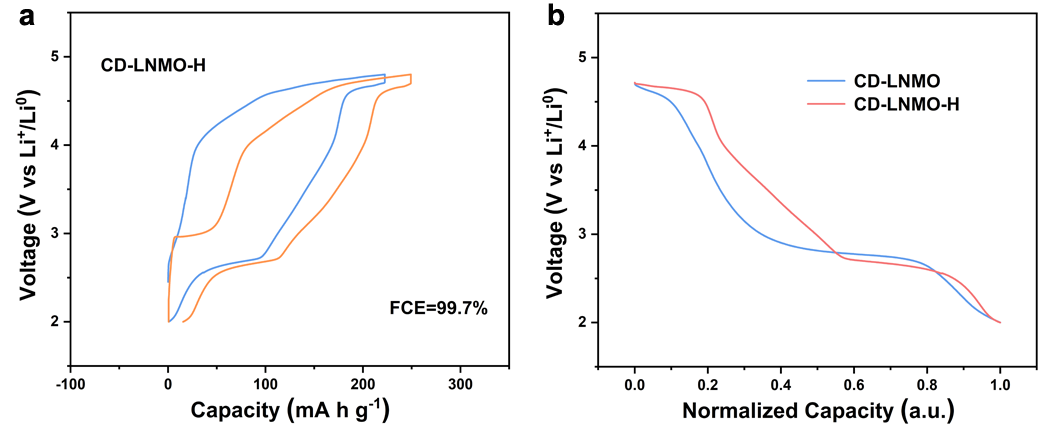


**Supplementary Figure 6 | Electrochemical behavior of CD-LNMO and CD-LNMO-H. a.** The first two cycles charge-discharge profile of CD-LNMO-H within 2.0–4.8 V, the tests are performed in Li metal coin cells with specific current of 100 mA g^-1^ at 25±5°C. **b.** Normalized discharge profile of CD-LNMO and CD-LNMO-H during the first cycle.


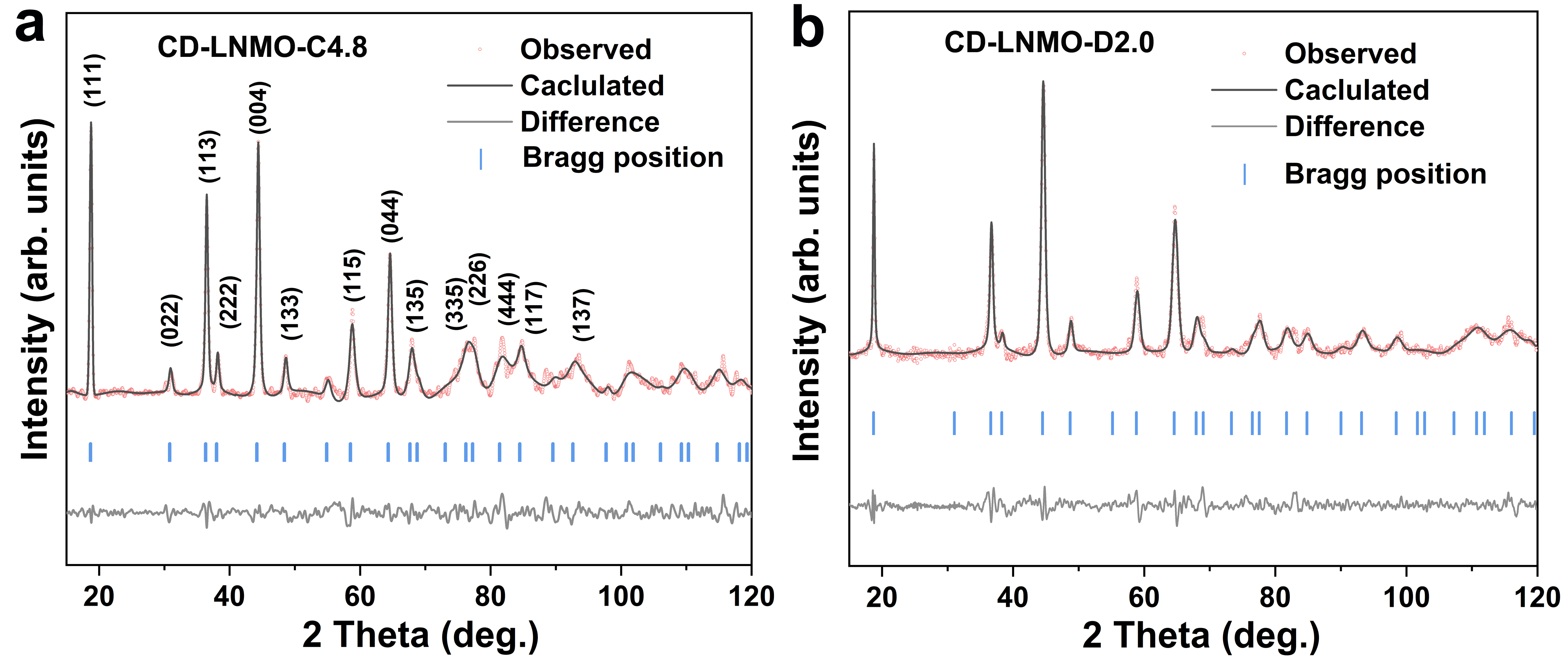


**Supplementary Figure 7 |** Crystal structure of CD-LNMO electrodes under different states in the initial cycle. **a,b**  SXRD patterns and the Rietveld reﬁnement of **(a)** fully-charged (charged to 4.8 V, denoted as CD-LNMO-C4.8) and **(b)** fully-discharged (discharged to 2.0 V, denoted as CD-LNMO-D2.0) sample. The goodness-of-fitting parameters *R*_wp_ are 4.68% and 4.28%, respectively. The tests are performed in Li metal coin cells with specific current of 100 mA g^-1^ at 25±5℃, the cells are dissembled immediately at target voltages in the first cycle.


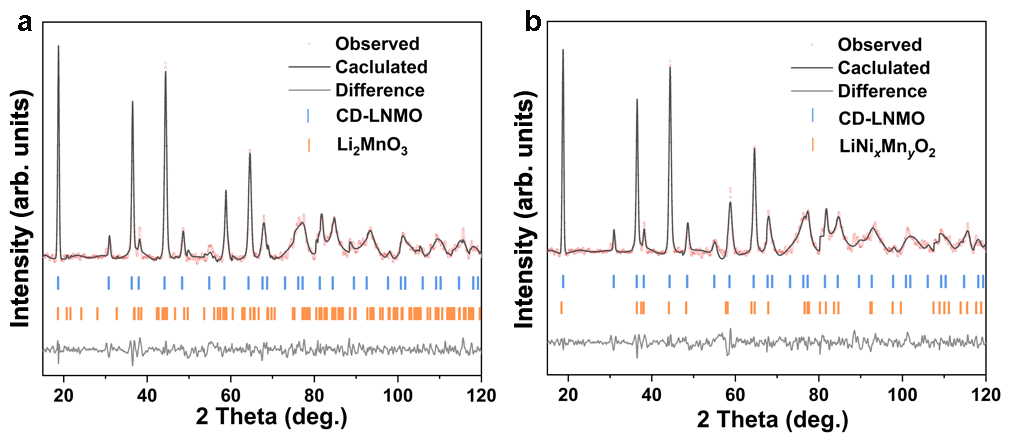


**Supplementary Figure 8 |** **S**XRD refinement of 4.8 V charged CD-LNMO electrodes based on the two-phase assumption. **a,b**  SXRD patterns and the Rietveld reﬁnement by considering (a) partially cationic disordered spinel phase and Li_2_MnO_3_ phase and (b) cationic disordered spinel phase and LiNi*_x_*Mn*_y_*O_2_ phase. The goodness-of-fitting parameters *R*_wp_ are 4.48% and 4.59%, respectively. The tests are performed in Li metal coin cells with specific current of 100 mA g^-1^ at 25±5℃, the cells are dissembled immediately at target voltages in the first cycle.


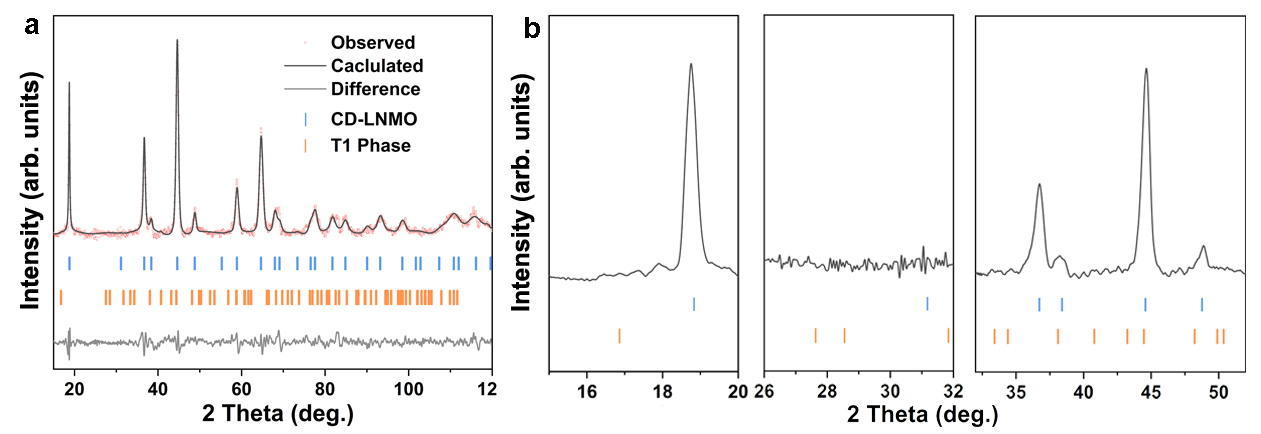


**Supplementary Figure 9 |** **S**XRD refinement of 2.0 V discharged CD-LNMO electrodes based on the two-phase assumption. **a.** The **S**XRD pattern and the refinement of 2.0 V discharged CD-LNMO electrodes by considering partially cationic disordered spinel phase and T1 phase. **b.** Highlighted regions in the **S**XRD pattern of 2.0 V discharged CD-LNMO electrodes. The goodness-of-fitting parameters *R*_wp_ is 4.26%. The tests are performed in Li metal coin cells with specific current of 100 mA g^-1^ at 25±5℃, the cells are dissembled immediately at target voltages in the first cycle.


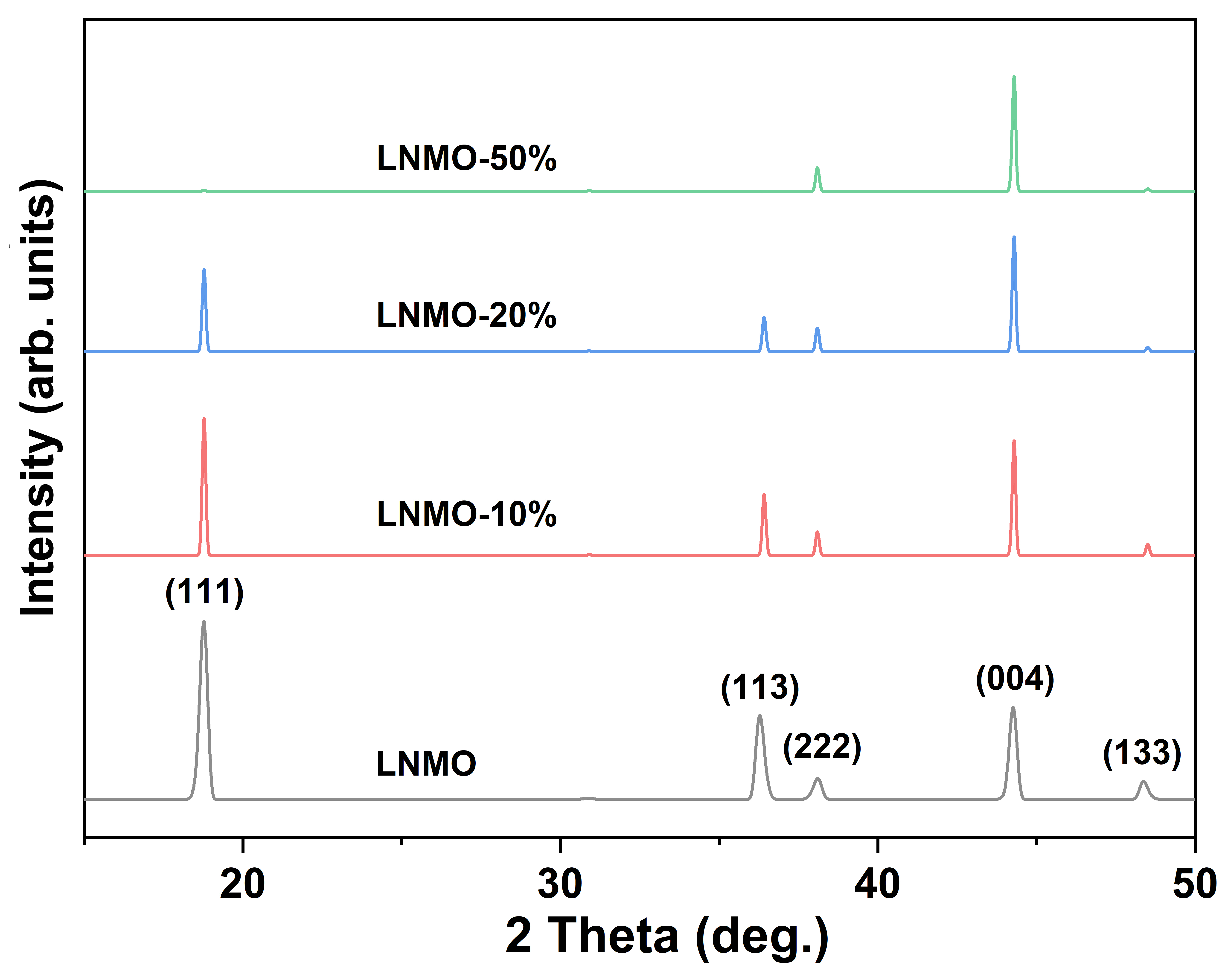


**Supplementary Figure 10 |** Simulated XRD pattern of LNMO with different degrees of cation disordering. LNMO, LNMO-10 at%, LNMO-20 at% and LNMO-50 at% are LiNi_0.5_Mn_1.5_O_4_ (space group of *Fd m*) crystal with 0 at%, 10 at%, 20 at% and 50 at% TM ions located in the 16c sites.


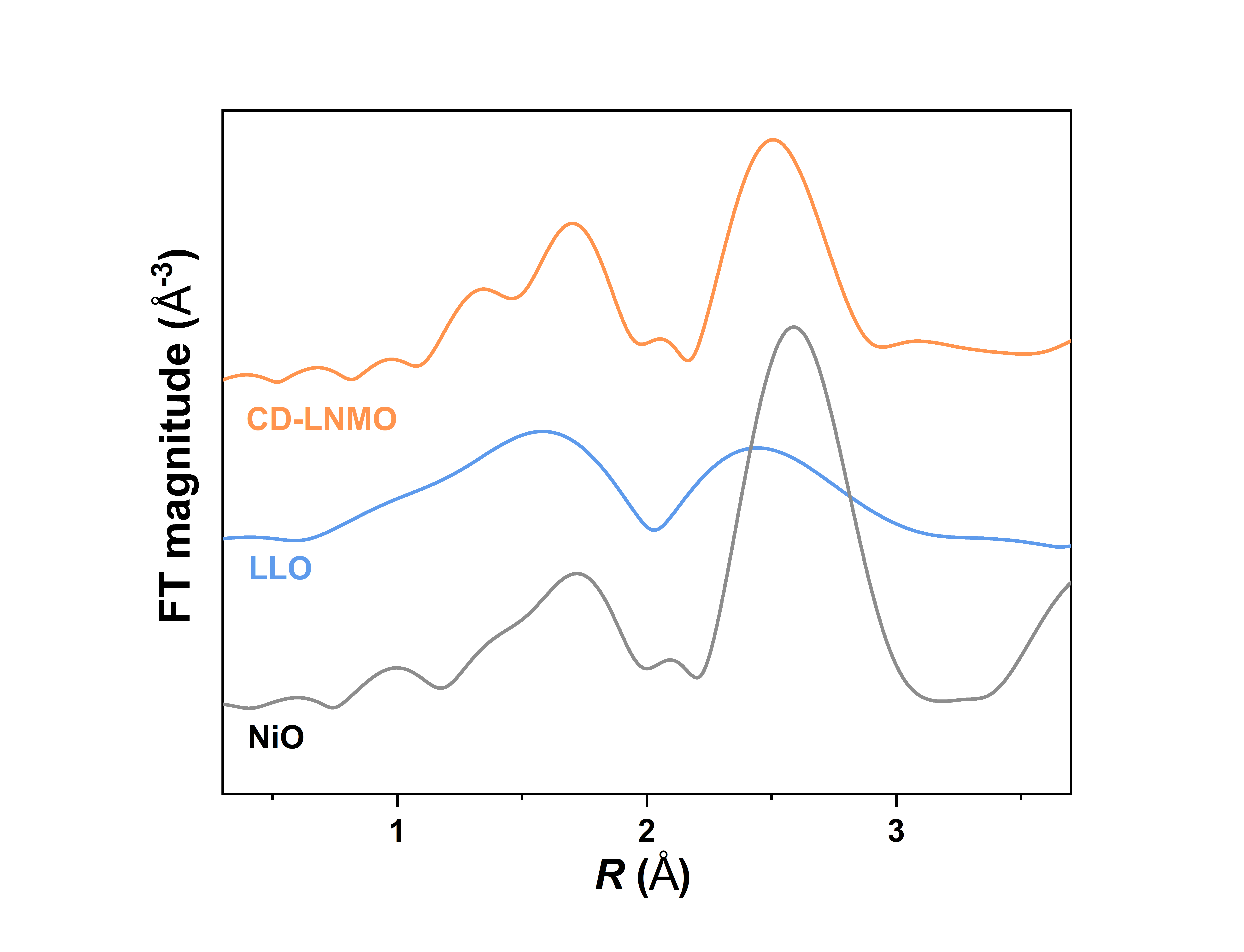


**Supplementary Figure 11 |** ***k*^3^-weighted Fourier transform magnitudes of Ni K-edge EXAFS spectra obtained from LLO and CD-LNMO powders.** The Ni K-edge EXAFS spectra of NiO are shown as reference.


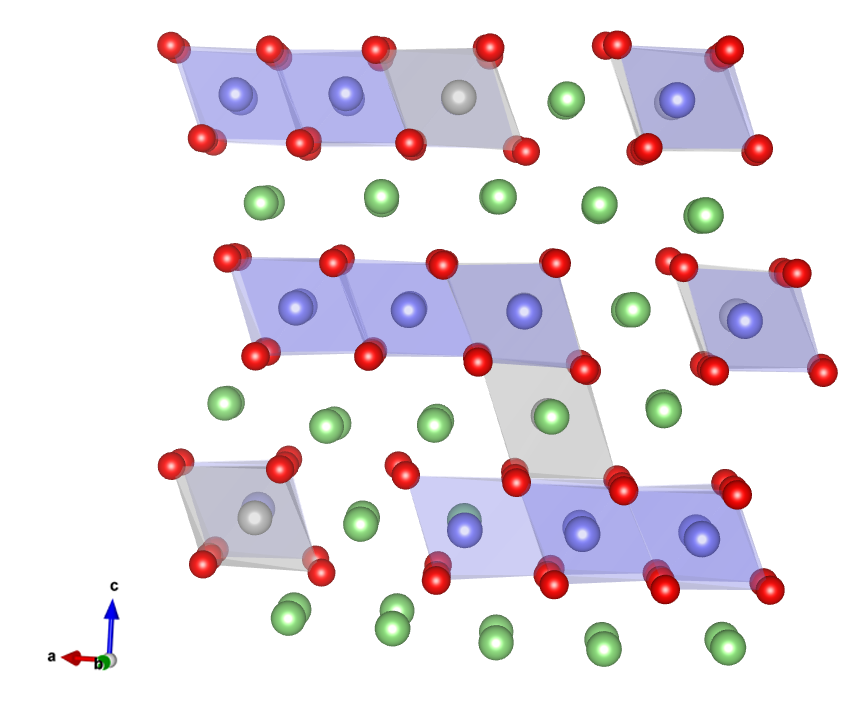


**Supplementary Figure 12 | Crystal structure of Li_1.2_Ni_0.2_Mn_0.6_O_2_ crystal with partially interlayer Li/TM mixing.** The Mn, Ni, Li ions and oxygen ions are shown as purple, blue, green and red balls, respectively. The MnO_6_ and NiO_6_ ligands are represented by blue and gray octahedrons, respectively.


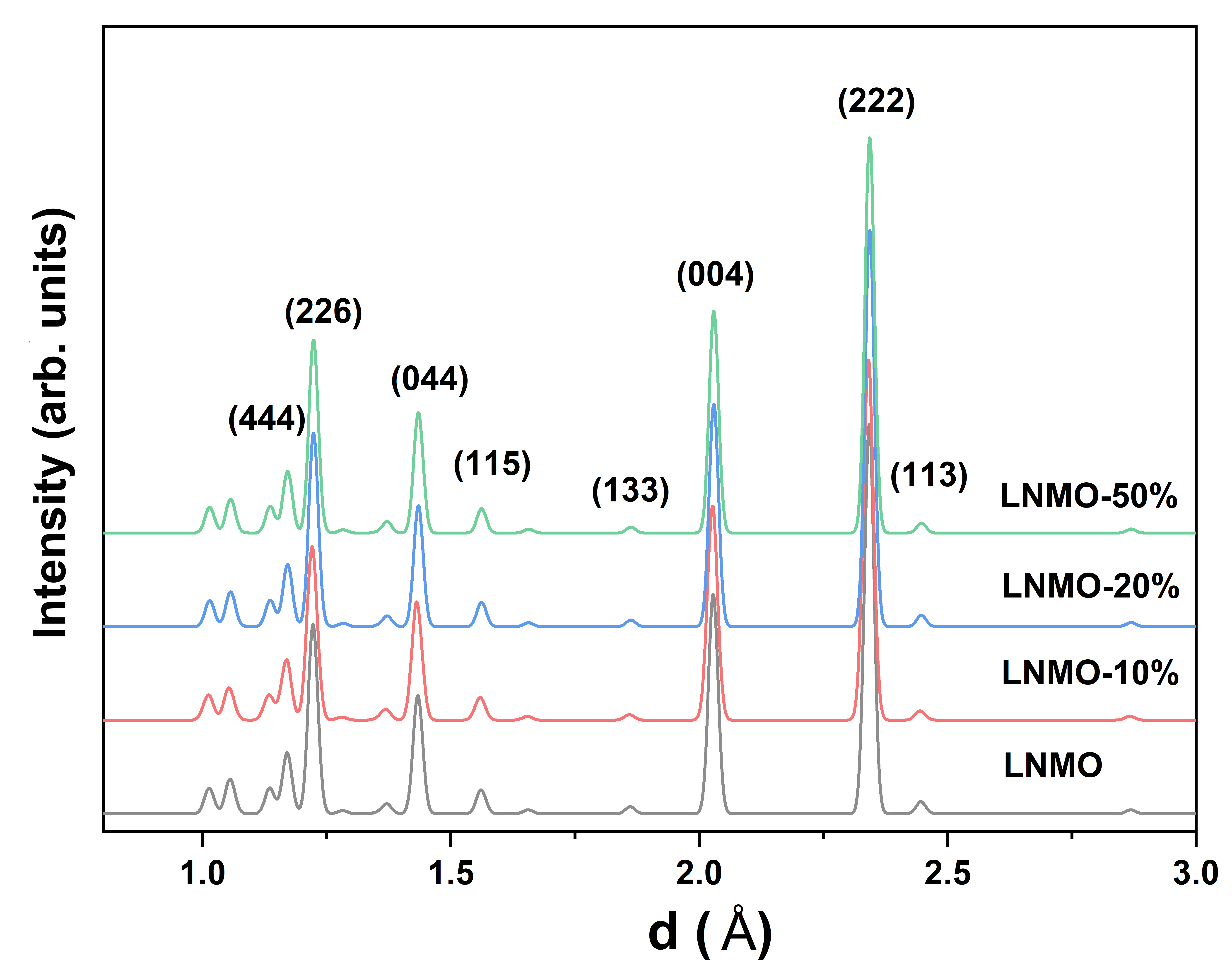


**Supplementary Figure 13 |** **Simulated ND pattern of LNMO with different degrees of cation disordering.** LNMO, LNMO-10 at%, LNMO-20 at% and LNMO-50 at% are LiNi_0.5_Mn_1.5_O_4_ (space group of *Fd m*) crystal with 0 at%, 10 at%, 20 at% and 50 at% TM ions located in the 16c sites.


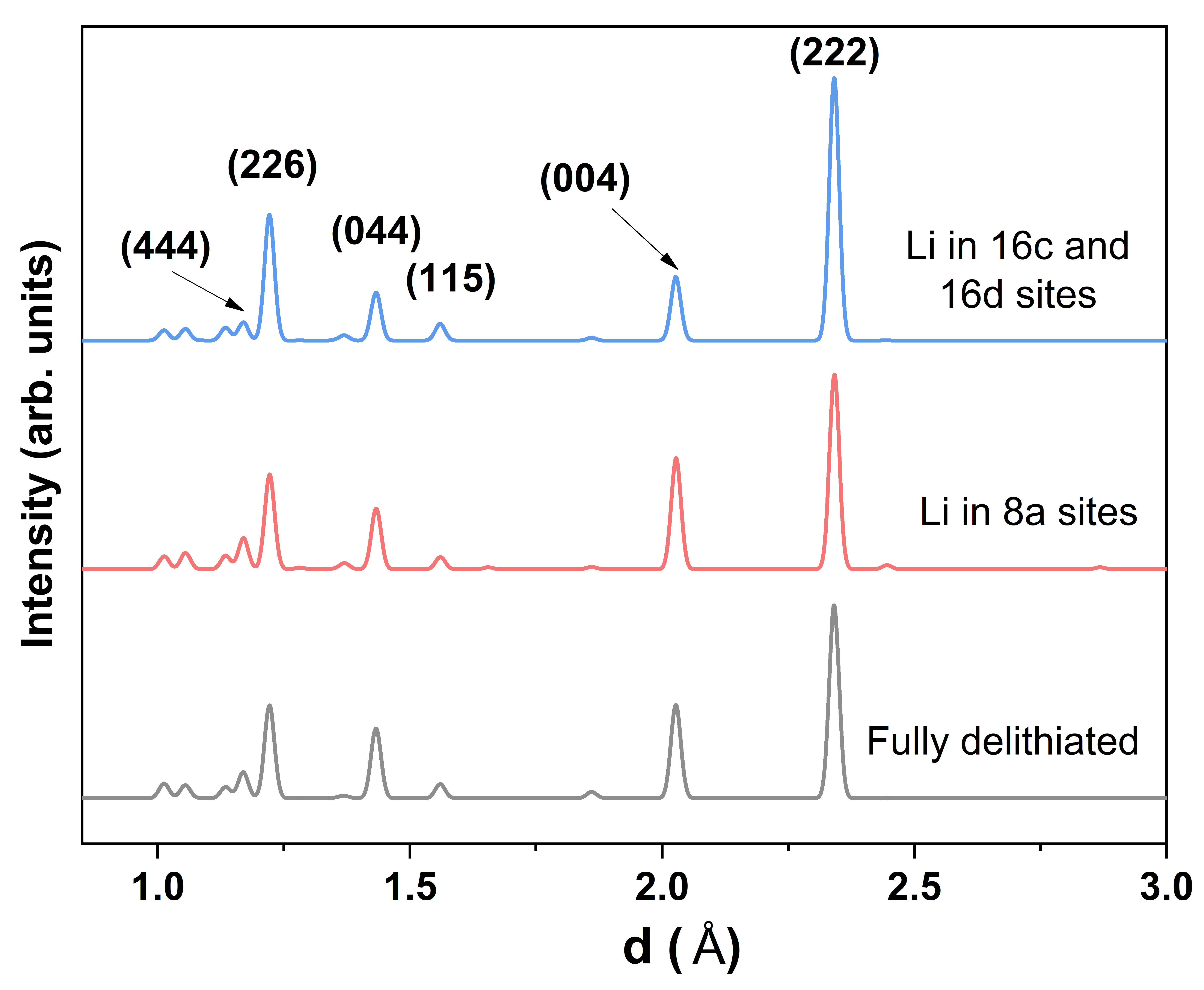


**Supplementary Figure 14 | Simulated ND patterns of CD-LNMO under different conditions.** Compared to the fully delithiated one, the insertion of Li into 8a tetrahedral sites of CD-LNMO (100 at% occupied by Li ions) results in a strengthened (004) reflection, while the insertion of Li in to 16c and 16d octahedral sites of CD-LNMO (100 at% occupied by Li and TM ions) results in a weakened (004) reflection.


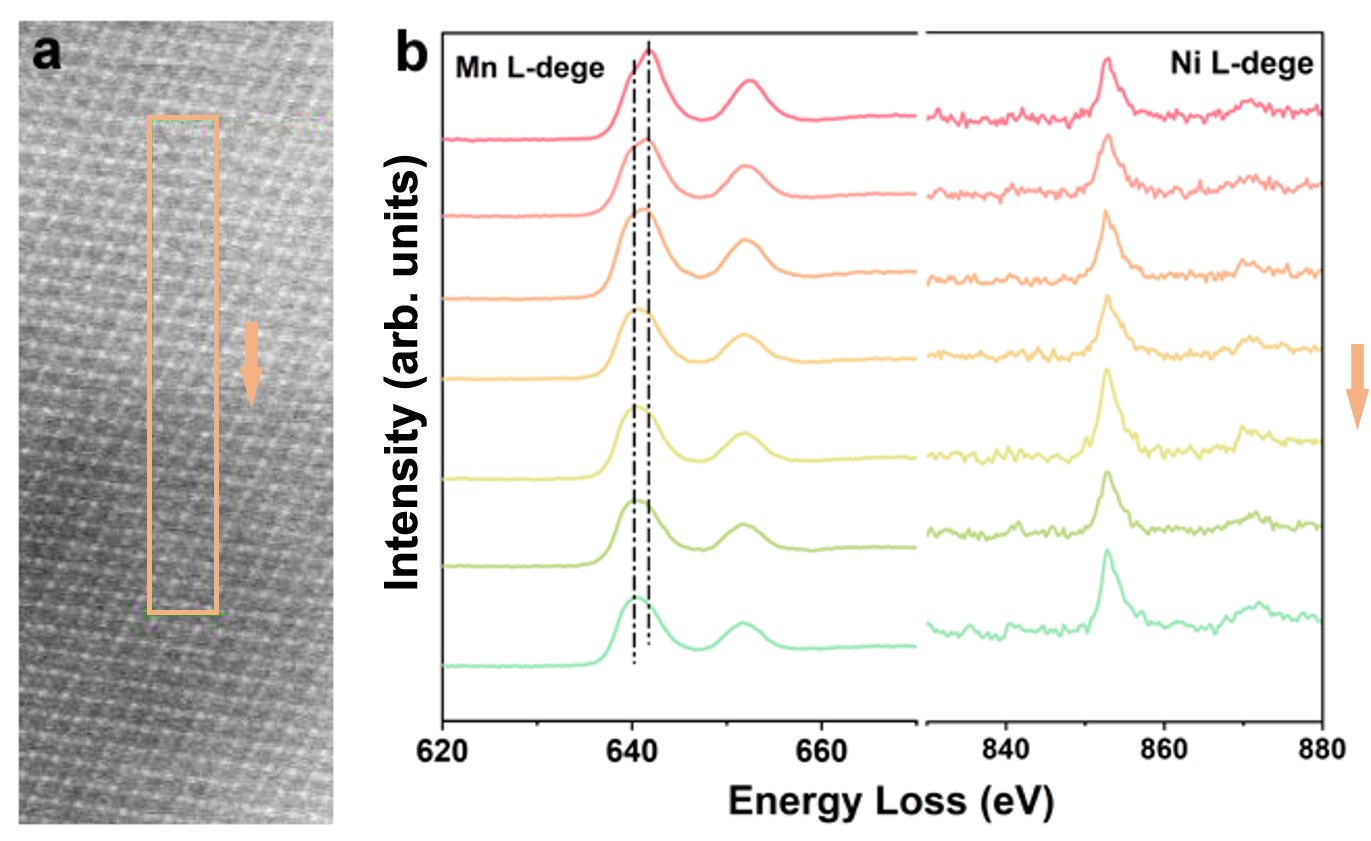


**Supplementary Figure 15 | Electron energy loss spectroscopy (EELS) of CD-LNMO powder. a.** HAADF-STEM image of EELS scan area. The scanned region is marked by an orange frame. **b.** Mn L-edge and Ni L-edge EELS spectra of the selected region. The orientation of the EELS scan is marked by orange arrows.


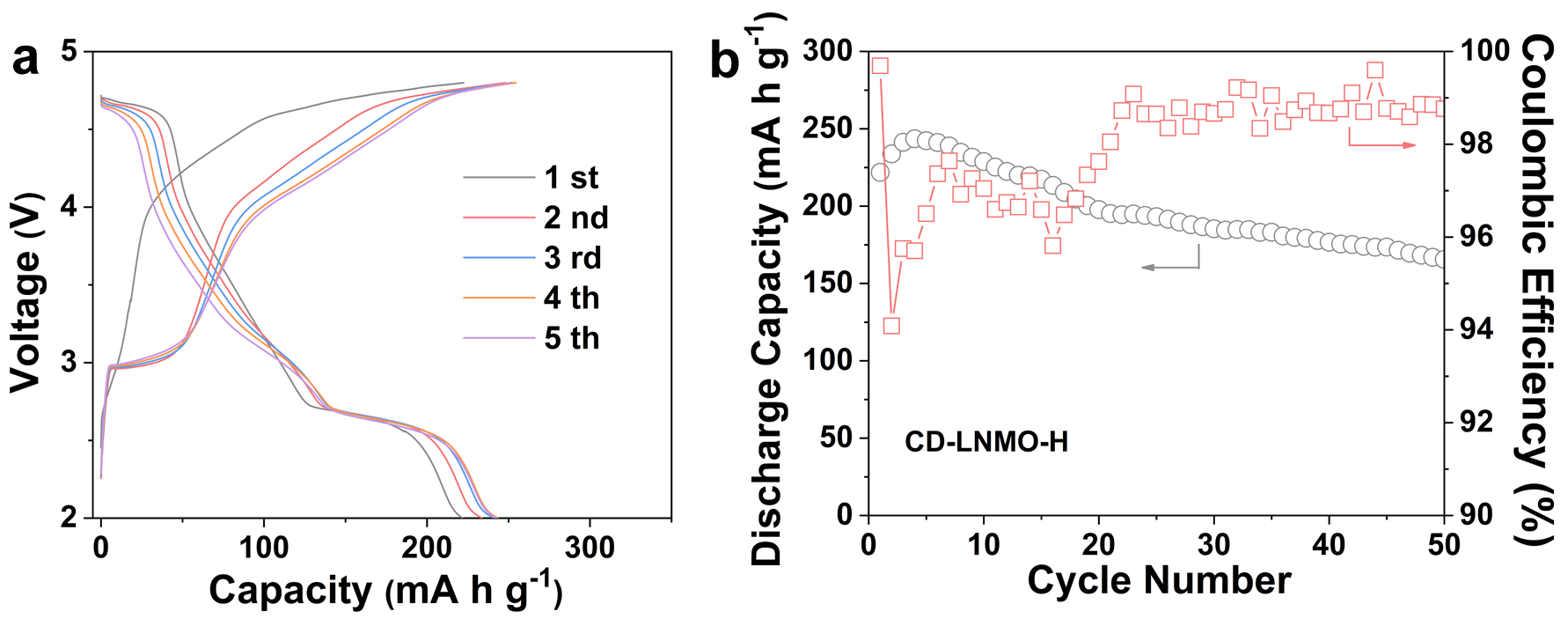


**Supplementary Figure 16 | Electrochemical performance of CD-LNMO-H. a.** The charge-discharge profile of CD-LNMO-H in the first five cycles within the work window of 2.0–4.8 V. **b.** The cycling stability and Coulombic efficiency of CD-LNMO-H within the work window of 2.0–4.8 V. The dark cycles and red squares indicate the discharge capacities and Coulombic efficiencies, respectively. The tests are performed in Li metal coin cells with specific current of 100 mA g^-1^ at 25±5℃.


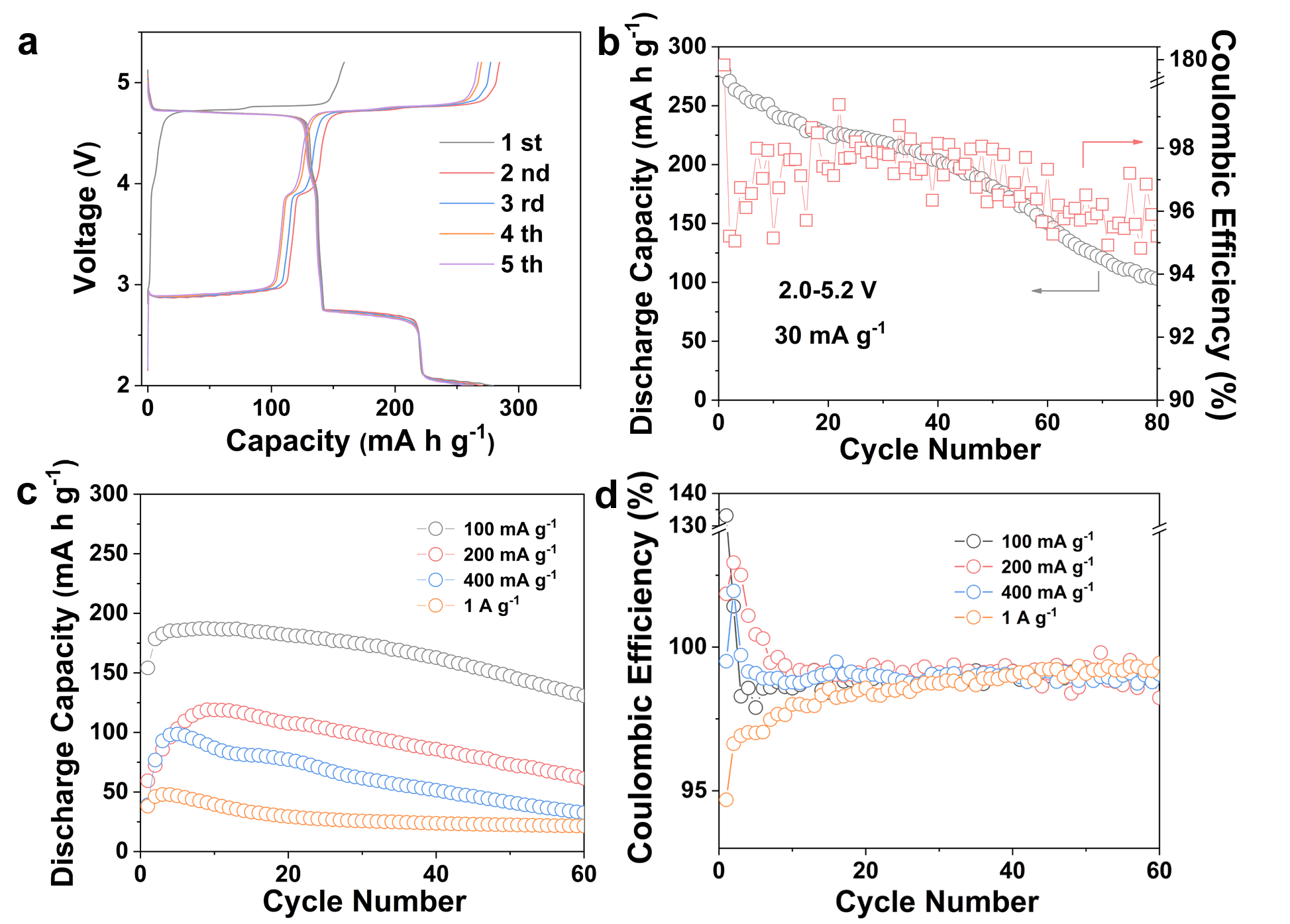


**Supplementary Figure 17 | Electrochemical performance of LNMO. a.** The charge-discharge profile of LNMO in the first five cycles within the work window of 2.0–5.2 V. (specific current is 30 mA g^-1^). **b.** The cycling stability and Coulombic efficiency of LNMO within the work window of 2.0–5.2 V. (specific current is 30 mA g^-1^). The dark cycles and red squares indicate the discharge capacities and Coulombic efficiencies, respectively. **c,d.** The cycling stability **(c)** and Coulombic efficiency **(d)** of LNMO under different specific currents. (work window of 2.0–4.8 V). The tests are performed in Li metal coin cells at 25±5℃.


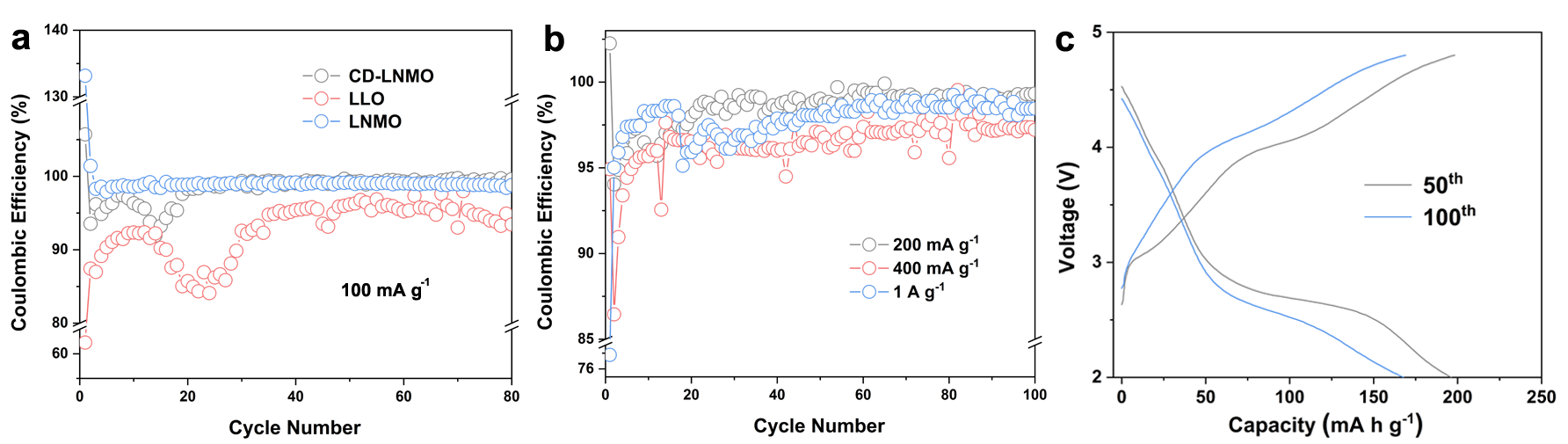
 **Supplementary Figure 18 | Electrochemical behavior of CD-LNMO, LLO and LNMO upon 100 cycles.** **a.** The Coulombic efficiencies of CD-LNMO, LLO and LNMO at a specific current of 100 mA g^-1^. **b.** The Coulombic efficiencies of CD-LNMO under different specific currents. **c.** The charge/discharge profiles at 50^th^ and 100^th^ cycles under 200 mA g^-1^. The tests are performed in Li metal coin cells within the work window of 2.0–4.8 V at 25±5℃.


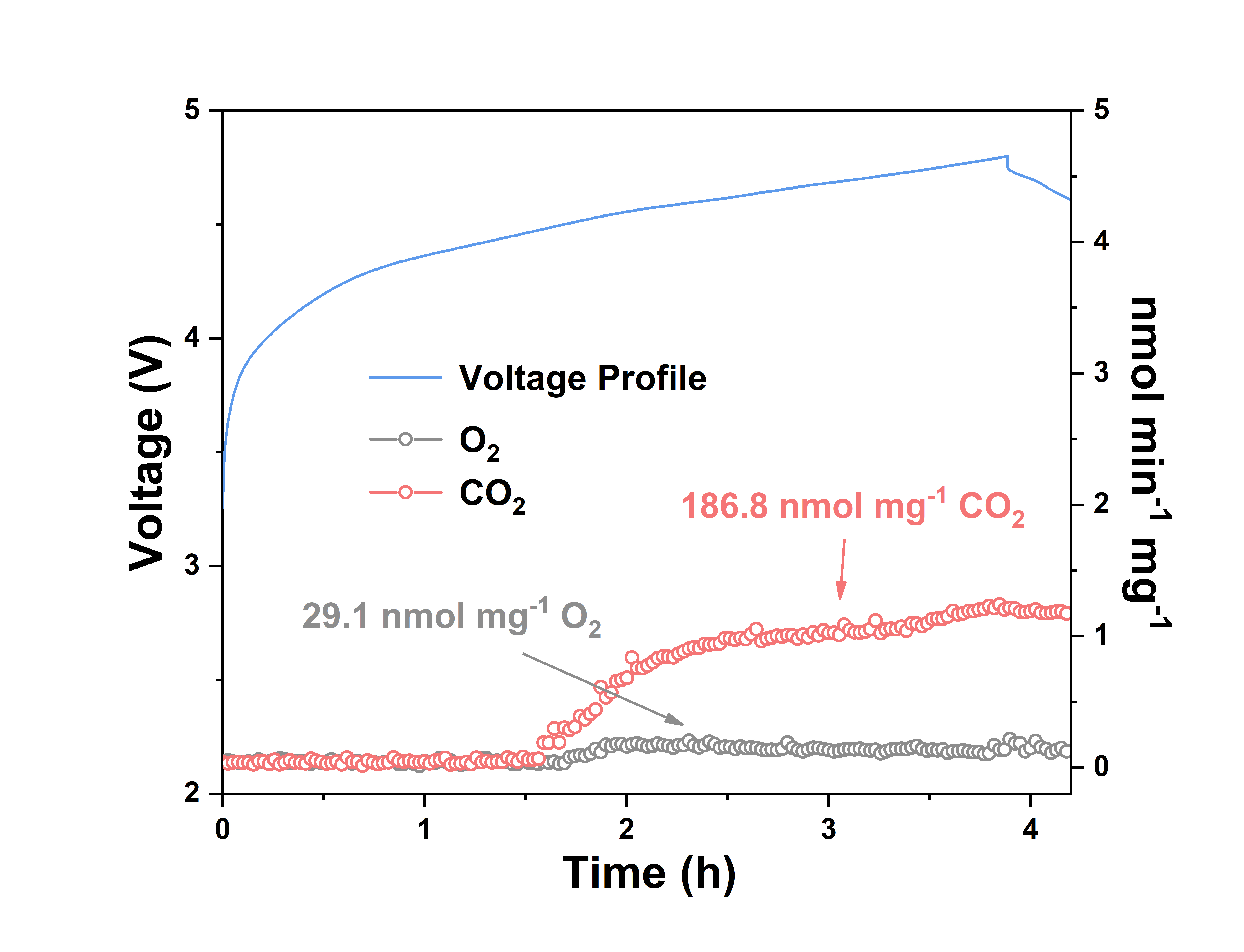


**Supplementary Figure 19 |** **Gas evolution analysis of CD-LNMO through operando differential electrochemical mass spectroscopy (DEMS).** The DEMS measurement is carried out with the specific current of 60 mA g^−1^, the signal of CO_2_ and O_2_ are shown as red and grey cycles, respectively. The CO_2_ evolution starts at ~4.5 V is mostly traced from the alkali carbonate decomposition.^1-3^


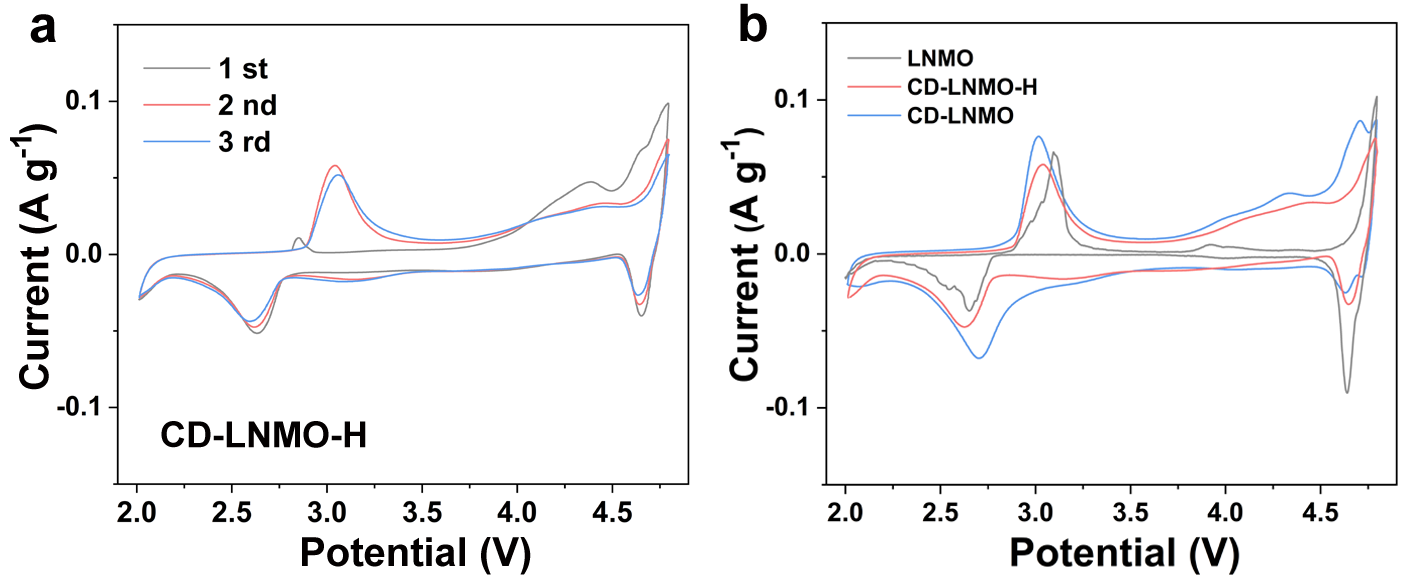


**Supplementary Figure 20 | Comparison of cyclic voltammetry (CV) curves. a.** The first three cycles CV curves of CD-LNMO-H. **b.** The second cycle CV curve of LNMO, CD-LNMO-H and CD-LNMO. The scan rate of each CV test is 0.05 mV s^–1^. The tests are performed in Li metal coin cells at 25±5℃, therefore the potential value is referred to the Li/Li^+^ redox couple.


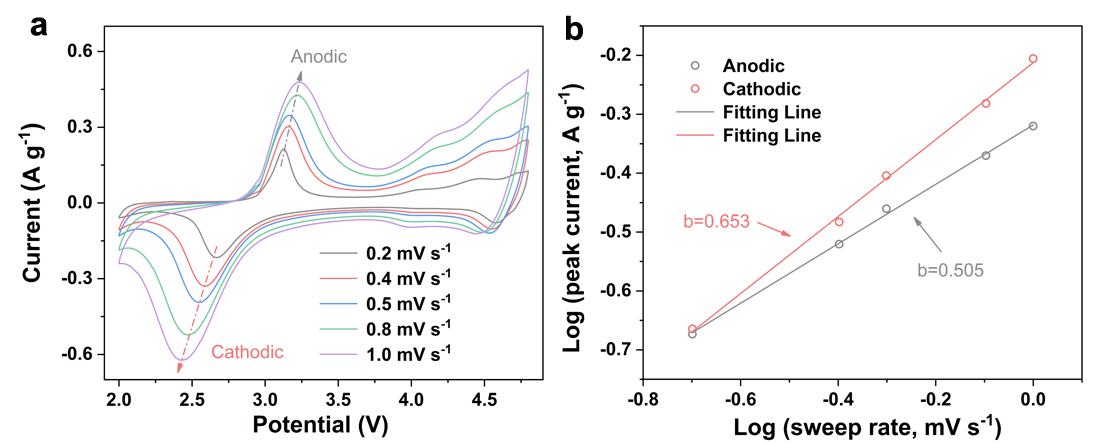


**Supplementary Figure 21 |** **Faradic and non-Faradaic charge storage mechanism in CD-LNMO-based electrodes. a.** Scan-rate depended CV with various scan rates from 0.2 to 1.0 mV s^-1^, **b.**  the plot of log *i* and log *v* under various scan rates. The *b* value value is the slop of fitted lines, which is generally utilized to analysis the Faradic and non-Faradaic behavior. The *b* value of 0.505 for the anodic redox indicates a diffusion-controlled faradaic process, while the *b* value of 0.653 for cathodic redox implies the co-existence of capacitive-controlled non-faradaic and diffusion-controlled faradaic process.^4^ The tests are performed in Li metal coin cells at 25±5℃, therefore the potential value is referred to the Li/Li^+^ redox couple.


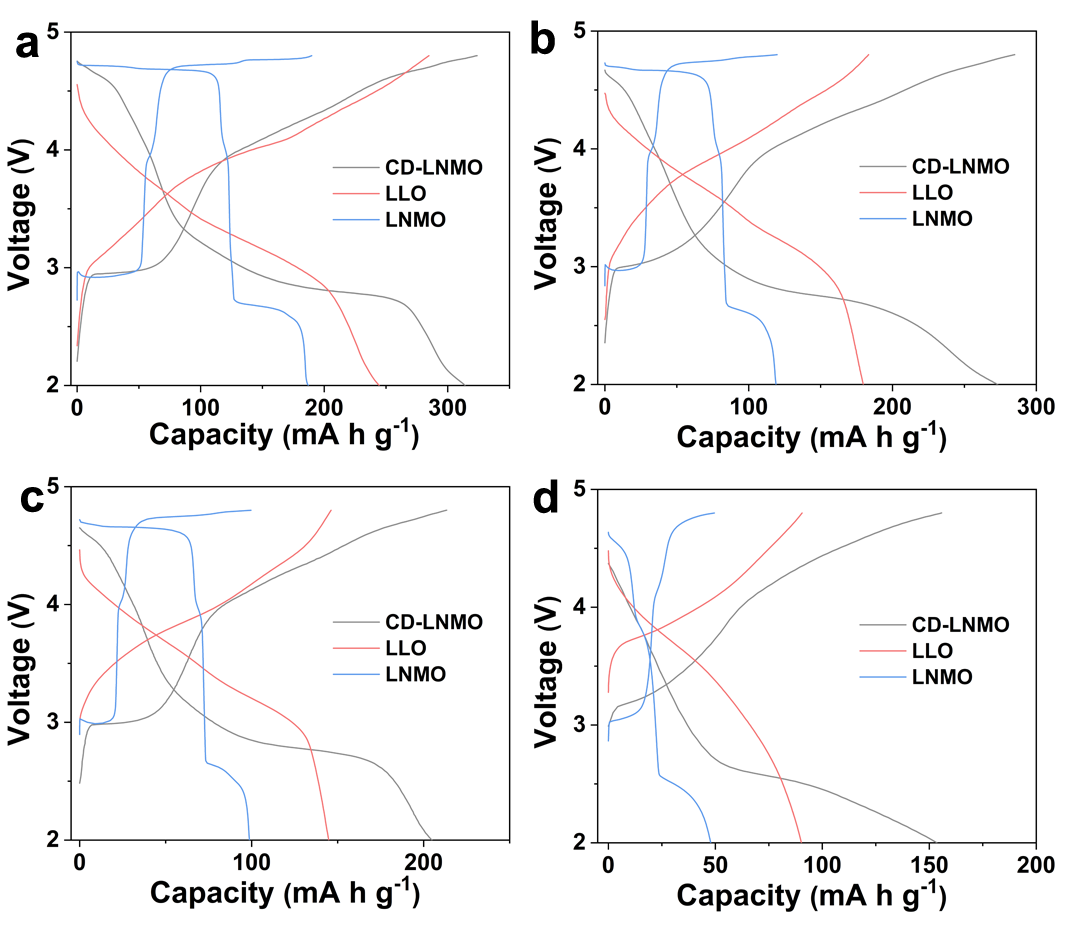


**Supplementary Figure 22 | The charge-discharge profile of CD-LNMO, LLO and LNMO under different specific currents. a-d.** The charge-discharge profile of CD-LNMO, LLO and LNMO under (a) 100 mA g^-1^, (b) 200 mA g^-1^, (c) 400 mA g^-1^ and (d) 1000 mA g^-1^. To circumvent the influence of capacity activation in initial cycles and have a reliable comparison, the charge-discharge profiles with the highest discharge capacities are compared (at 100 mA g^-1^, the profile at 9^th^, 27^th^ and 9^th^ cycle of CD-LNMO, LLO and LNMO, respectively; at 200 mA g^-1^, the profile at 5^th^, 13^th^ and 9^th^ cycle of CD-LNMO, LLO and LNMO, respectively; at 400 mA g^-1^, the profile at 11^th^, 22^th^ and 5^th^ cycle of CD-LNMO, LLO and LNMO, respectively; at 1 A g^-1^, the profile at 14^th^, 19^th^ and 3^th^ cycle of CD-LNMO, LLO and LNMO, respectively). The tests tests are performed in Li metal coin cells within 2.0–4.8 V at 25±5℃.


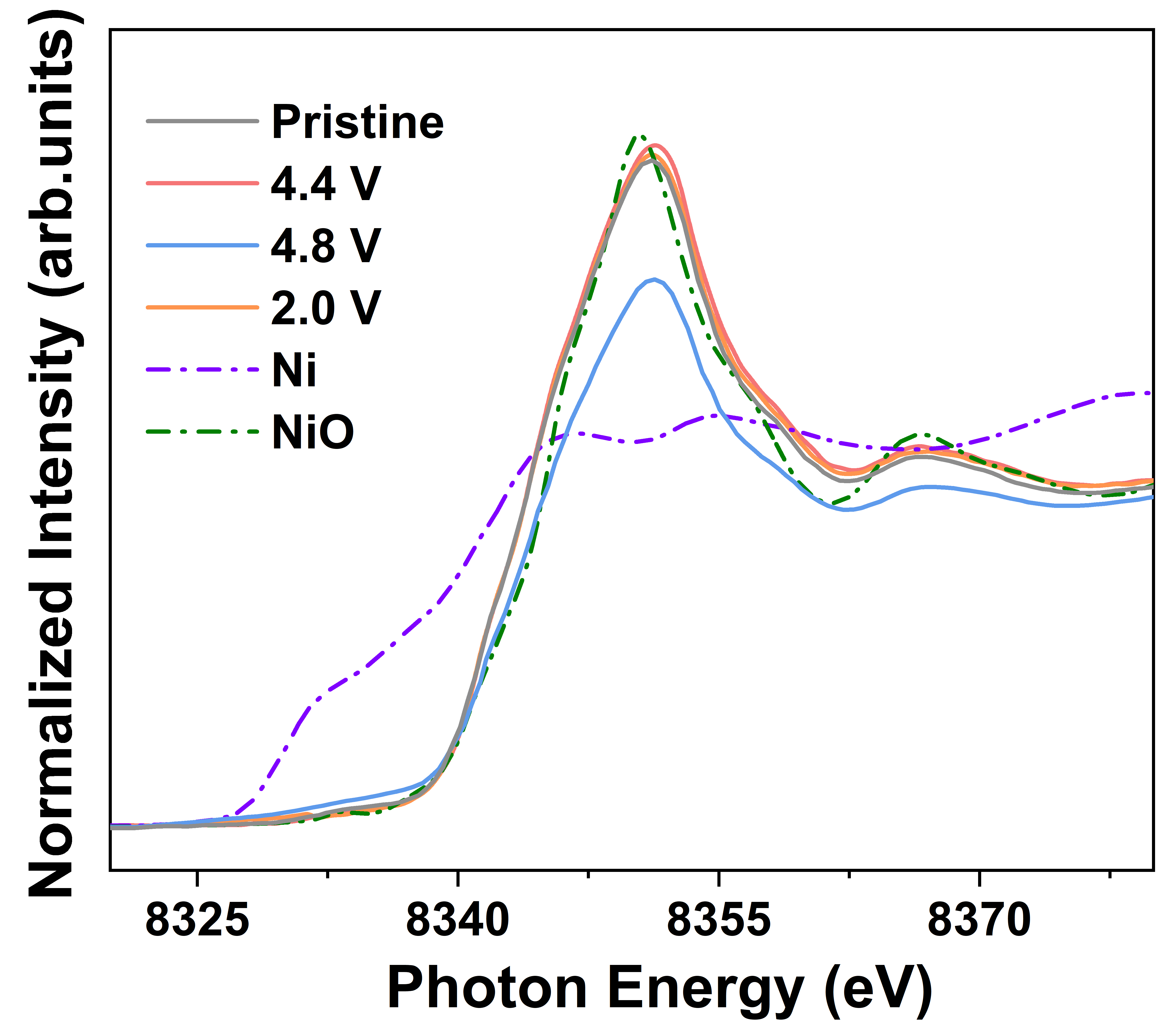


**Supplementary Figure 23 |** **Electronic evolution of Ni during the initial cycle.** Normalized ex situ Ni K-edge XANES spectra obtained from CD-LNMO electrodes at different voltages of the first cycle. The Ni K-edge XANES spectra of Ni foil and NiO are shown as references. The 4.4 V and 4.8 V are charged states, while the 2.0 V is the discharge state, the cells are dissembled immediately at target voltages in the first cycle. The tests are performed in Li metal coin cells with specific current of 100 mA g^-1^ at 25±5℃.


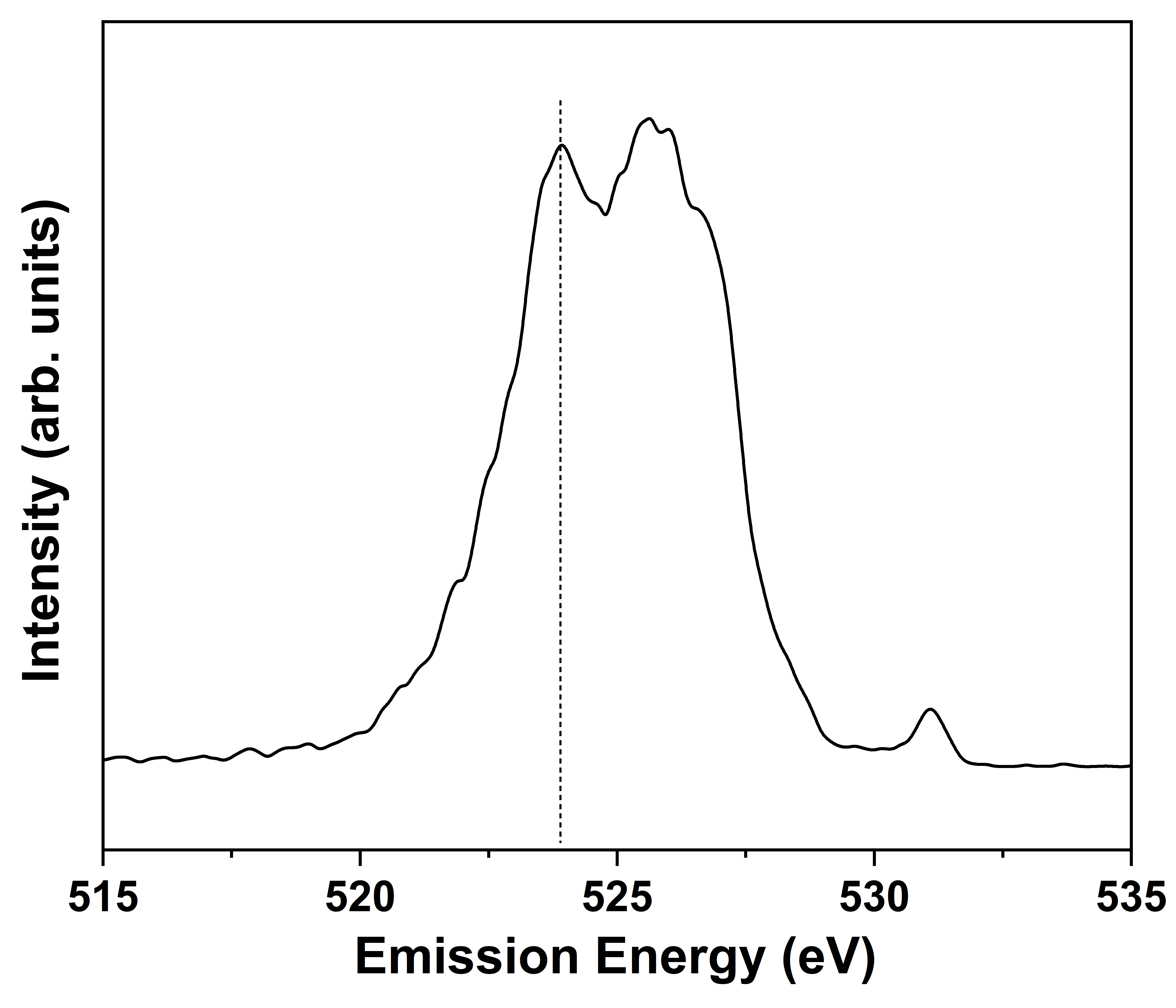


**Supplementary Figure 24 | mRIXS cuts of** 4.**8V charged electrodes obtained with** **specific current of 100 mA g^-1^ at an excitation energy of 531 eV.** The O*^n^*^-^ (*n*<2) feature peak is marked by dash line.


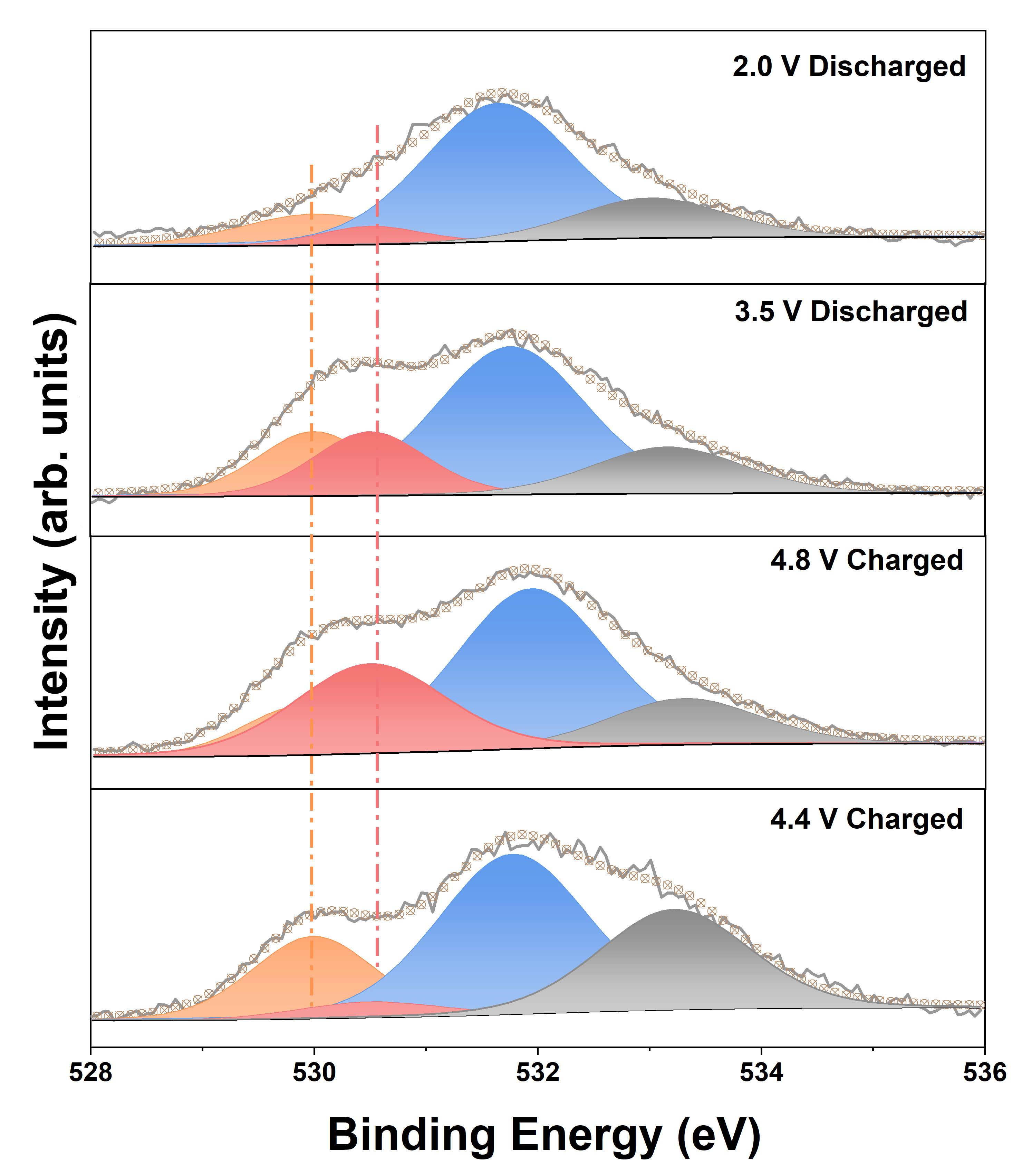


**Supplementary Figure 25 | O 1*s* XPS spectra of CD-LNMO at different charge states during initial cycle**. The electrodes are etched by Ar for 30s to remove the surface adsorbed spices. The fitting was performed by considering four probable oxidation states of O: crystal lattice O^2-^ (529.5–530.0 eV); peroxo-like O-O pairs or oxygen with localized electron holes O*^n^*^-^ (~530.5 eV); deposited oxygenated species (531–532 eV) and electrolyte oxidation (533~534 eV). The tests are performed in Li metal coin cells with specific current of 100 mA g^-1^ at 25±5℃, and cells are dissembled immediately at target voltages in the first cycle.


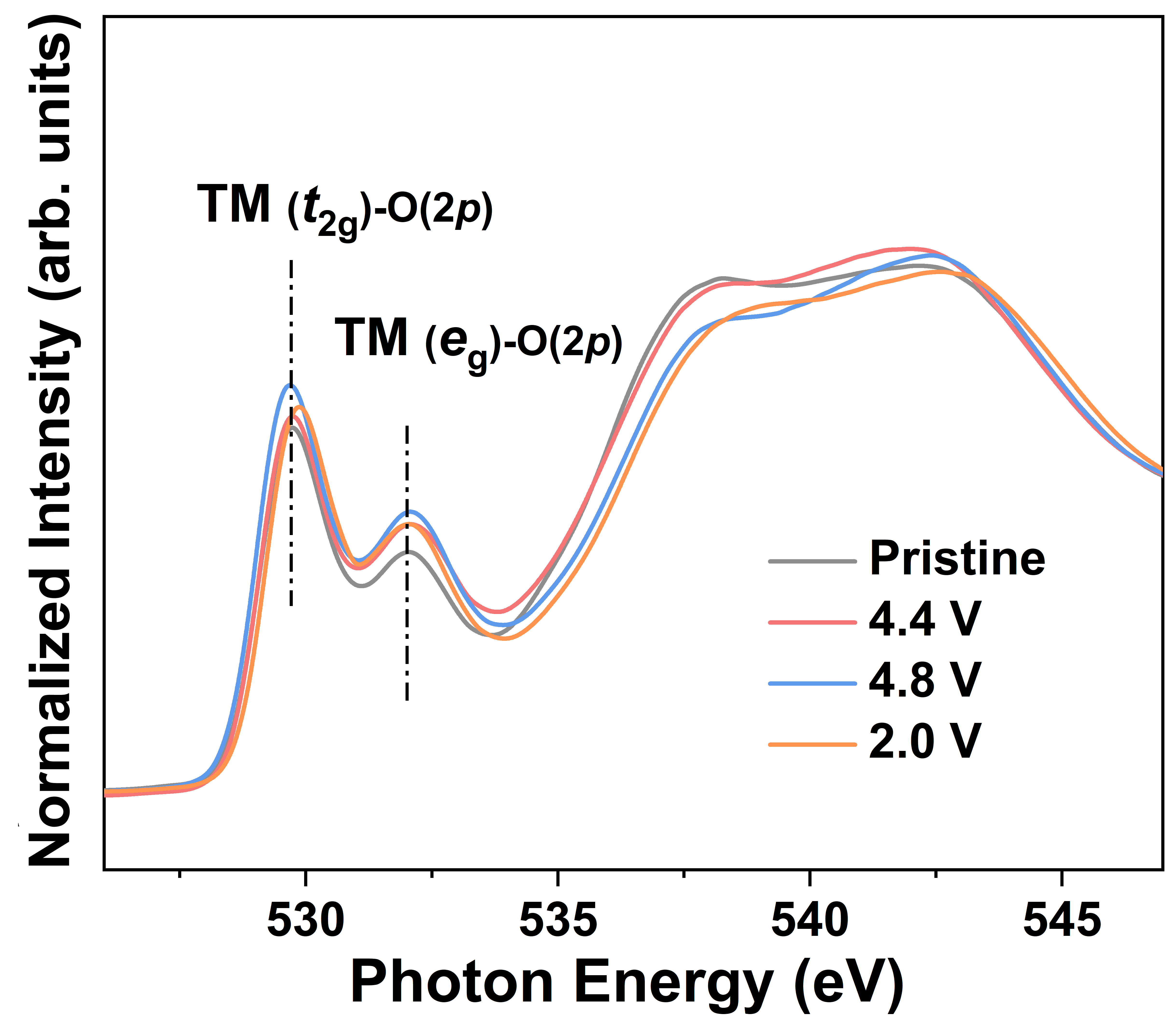


**Supplementary Figure 26 | Electronic evolution of O during the initial cycle.** Normalized ex situ O K-edge sXAS spectra obtained from CD-LNMO electrodes at different voltages during the first cycle. The 4.4 V and 4.8 V are charged states, while the 2.0 V is the discharge state, the cells are dissembled immediately at target voltages in the first cycle. The tests are performed in Li metal coin cells with specific current of 100 mA g^-1^ at 25±5℃.


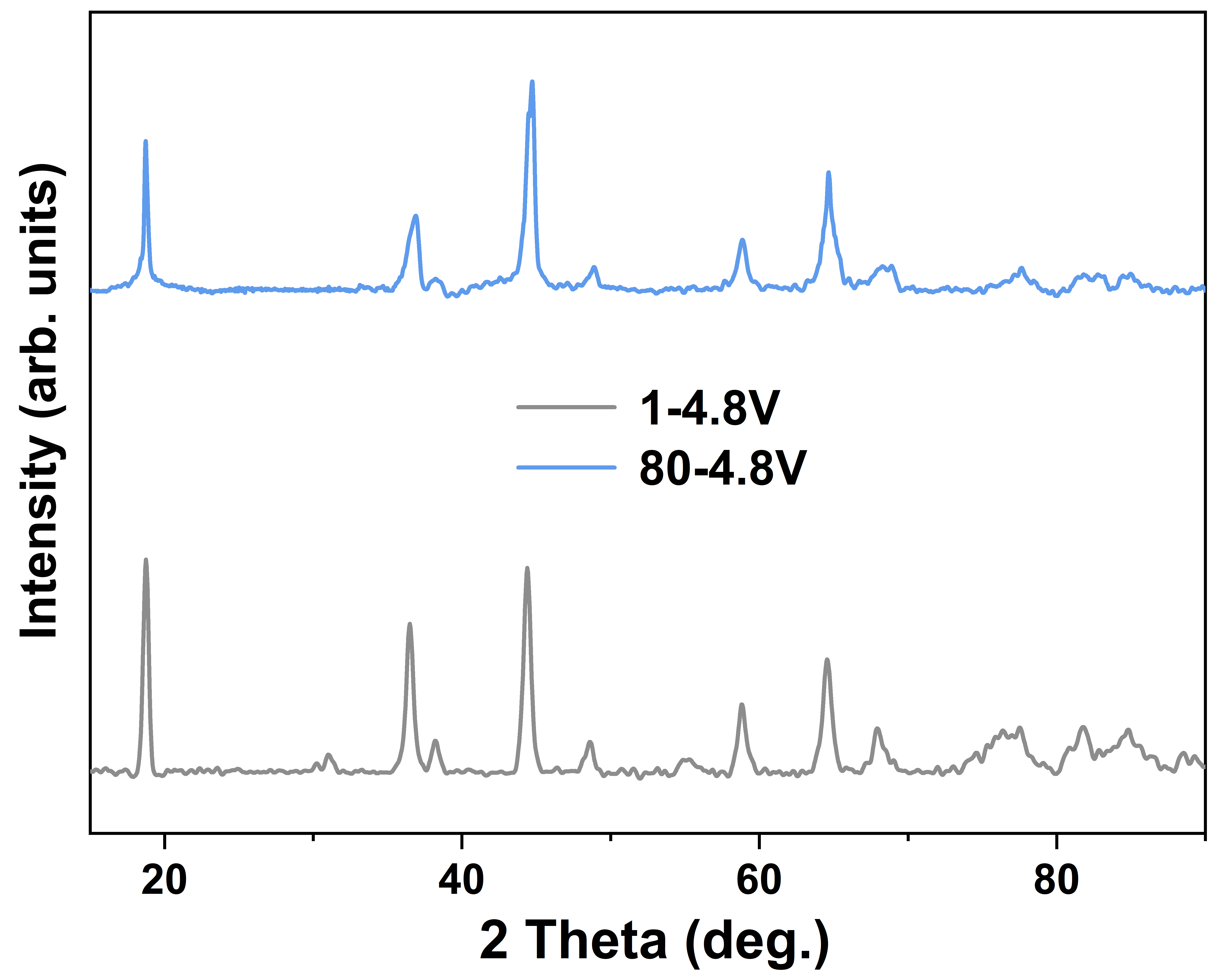


**Supplementary Figure 27 | Ex situ SXRD measurements on CD-LNMO.** The electrodes charged to 4.8 V in the initial cycle and 80^th^ cycle is labelled as 1–4.8V and 80–4.8V, respectively. The tests are performed in Li metal coin cells with specific current of 100 mA g^-1^ at 25±5℃, and cells are dissembled immediately at target voltages.


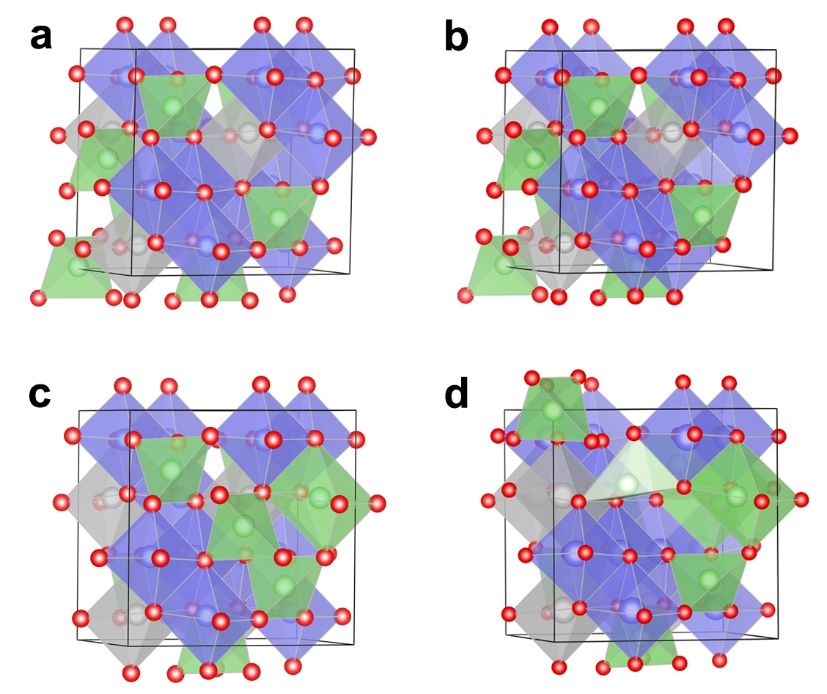


**Supplementary Figure 28 | Crystal structures of LNMO before and after structural evolutions. a-d.** Structures of (a) LNMO, (b) CD-LNMO-1, (c) Li-rich LNMO and (d) CD-LNMO. The LiO_6_, MnO_6_ and NiO_6_ ligands are represented by green, blue and gray octahedrons, respectively, and LiO_4_ ligands are shown as green tetrahedrons. The Mn, Ni, Li, and O ions are shown as purple, blue, green, and red balls, respectively.


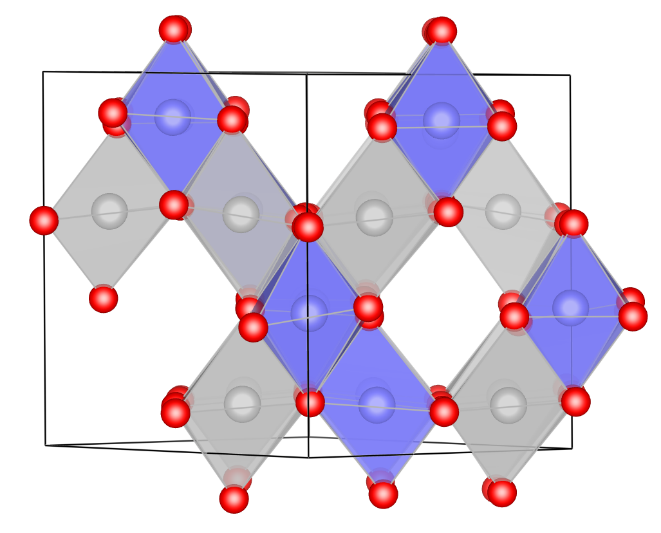


**Supplementary Figure 29 | Crystal structures of fully delithiated CD-LNMO.** The MnO_6_ and NiO_6_ ligands are represented by blue and gray octahedrons, respectively. The Mn, Ni and O ions are shown as purple, blue and red balls, respectively.

**Supplementary Table 1 | Relative element ratio measured by ICP.** The concentration is calculated by normalizing the content of Mn to 0.6 for both samples. The measurement errors are shown in the brackets.

| Sample | Li | Mn | Ni | Li/(Mn+Ni) |
| --- | --- | --- | --- | --- |
| LLO | 1.246(7) | 0.600(0) | 0.192(3) | 1.573(5) |
| CD-LNMO | 0.728(9) | 0.600(0) | 0.156(2) | 0.963(9) |

**Supplementary Table 2 | Structural information obtained from the SXRD and ND reﬁnements.** CD-LNMO-C4.8 and CD-LNMO-D2.0 represent the fully-charged (4.8 V) and fully-discharged CD-LNMO (2.0 V), respectively. The tests are performed in Li metal coin cells with specific current of 100 mA g^-1^ at 25±5℃, and cells are dissembled immediately at target voltages.

| Atom | Wyckoff symbol | *x* | *y* | *z* | Occupancy in CD-LNMO-C4.8 | Occupancy in CD-LNMO-D2.0 |
| --- | --- | --- | --- | --- | --- | --- |
| Li1 | 8a | 0.125 | 0.125 | 0.125 | 0.09(3) | 0.19(1) |
| Li2 | 16d | 0.5 | 0.5 | 0.5 | 0.00(3) | 0.25(9) |
| Li3 | 16c | 0 | 0 | 0 | 0.00(4) | 0.59(2) |
| Ni1 | 16d | 0.5 | 0.5 | 0.5 | 0.14(4) | 0.13(5) |
| Mn1 | 16d | 0.5 | 0.5 | 0.5 | 0.54(9) | 0.52(9) |
| Ni2 | 16c | 0 | 0 | 0 | 0.02 (9) | 0.04(0) |
| Mn2 | 16c | 0 | 0 | 0 | 0.09(2) | 0.11(6) |
| O1 | 32e | 0.259(4) | 0.259(4) | 0.259(4) | 1 | 1 |

**Supplementary Table 3 |** **The intensity ratio of the (004) and (222) reflections (*I*_(004)/(222)_) in the simulated and experimental ND patterns.** The tests are performed in Li metal coin cells with specific current of 100 mA g^-1^ at 25±5℃, and cells are dissembled immediately at target voltages.

| Samples | *I*_(004)/(222)_ |  |
| --- | --- | --- |
| 4.8 V charged CD-LNMO (Experimental) | | 0.45(8) |
| 2.0 V discharged CD-LNMO (Experimental) | | 0.31(8) |
| Fully delithiated CD-LNMO (Simulated) | | 0.48(9) |
| CD-LNMO with Li in 8a sites (Simulated) | | 0.57(3) |
| CD-LNMO with Li in 16c and 16d sites (Simulated) | | 0.24(0) |

**Supplementary Table 4 | Comparison of electrochemical performance of Li*_x_*TM*_y_*O_2_ (TM=Ni, Mn, Co, *etc*) cathodes. All the specific energies are calculated based on the positive electrode’s active material.**

| Li*_x_*TM*_y_*O_2_ Cathodes | Reversible capacity (mA h g^-1^) | Cycling stability | Specific energy  (Wh kg^-1^) | Refs. |
| --- | --- | --- | --- | --- |
| Li_1.2_Mn_0.2_Ti_0.4_Cr_0.2_O_2_ | 257  (under 20 mA g^-1^) | 200 mA h g^-1^ after 20 cycles | 790  (under 20 mAhg^−1^) | ^5^ |
| O2-Li*_x_*(Li_0.2_Ni_0.2_Mn _0.6_)O_2_ (*x* ≈ 0.83) | 235  (under 5mA g^−1^) | 175 mA h g^-1^ after 20 cycles | 727  (under 5mA g^−1^) | ^6^ |
| Li_1.3_Nb_0.3_Mn_0.4_ O_2_ | 300  (under 10mA g^−1^) | ~175 mA h g^-1^ after 20 cycles | 950  (under 10mA g^−1^) | ^7^ |
| Li_4_Mn_2_O_5_ | 355  (under 6 mA g^-1^) | 240 mA h g^-1^ after 8 cycles | 953  (under 6 mA g^−1^) | ^8^ |
| Li_1.2_Mn_0.6_Nb_0.2_O_2_ | 255  (under 20 mA g^-1^) | ~200 mA h g^-1^ after 20 cycles | -- | ^9^ |
| Li_1.2_Mn_0.4_Ti_0.4_O_2_ | ~260  (under 10 mA g^−1^) | ~230 mA h g^-1^ after 10 cycles | -- | ^10^ |
| Ta-doped Li[Ni_0.90_Co_0.09_ Ta_0.01_]O_2_ | 237  (under 20 mA g^−1^) | 220 mA h g^-1^ after 100 cycles | -- | ^11^ |
| Li_5_FeO_4_ | ~190  (under 19 mA g^-1^) | ~170 mA h g^-1^ after 25 cycles | -- | ^12^ |
| **CD-LNMO** | **314.1**  **(under 100 mA g^-1^)** | **179 mA h g^-1^ after 80 cycles** | **999.3**  **(Under 100 mA g^−1^)** | **this work** |

Test conditions:

Li_1.2_Mn_0.2_Ti_0.4_Cr_0.2_O_2_: The material is tested in Li metal coin cells, with 0.2 mL 1 M LiPF_6_ in EC/DMC electrolyte. The weight ratio of active material in cathode is 70%, and the mass loading is 3 mg cm^−2^. The work window is 1.5-4.8V. The highest discharge capacity is achieved in the first cycle, and the capacity retention is ~78% in 20 cycles.

O2-Li*_x_*(Li_0.2_Ni_0.2_Mn _0.6_)O_2_ (*x* ≈ 0.83): The material is tested in Li metal coin cells, with 1 M LiPF_6_ in EC/DMC electrolyte. The weight ratio of active material in cathode is 80%, and the work window is 2.0-4.8V. The highest discharge capacity is achieved in the first cycle, and the capacity retention is ~74% in 20 cycles.

Li_1.3_Nb_0.3_Mn_0.4_O_2_: The material is tested in Li metal Swagelok cells at 50 ℃, with 1 M LiPF_6_ in EC/DMC electrolyte. The weight ratio of active material in cathode is 72%, and the work window is 1.5-4.8V. The highest discharge capacity is achieved in the first cycle, and the capacity retention is ~58% in 20 cycles.

Li_4_Mn_2_O_5：_The material is tested in Li metal coin cells, with 1 M LiPF_6_ in EC/EMC electrolyte. The weight ratio of active material in cathode is 72%, and the mass loading is 10-15 g cm^−1^. The work window is 1.2-4.4V. The highest discharge capacity is achieved in the first cycle, and the capacity retention is ~68 % in 8 cycles.

Li_1.2_Mn_0.6_Nb_0.2_O_2_: The material is tested in Li metal coin cells, with 1 M LiPF_6_ in EC/DMC electrolyte. The weight ratio of active material in cathode is 70%, and the mass loading is ~4 mg cm^−2^.The work window is 1.5-4.8V. The highest discharge capacity is achieved in the first cycle, and the capacity retention is ~78 % in 20 cycles.

Li_1.2_Mn_0.4_Ti_0.4_O_2_: The material is tested in Li metal coin cells, with 1 M LiPF_6_ in EC/EMC electrolyte. The weight ratio of active material in cathode is 70%. The work window is 1.5-4.7V. The highest discharge capacity is achieved in the first cycle, and the capacity retention is ~88 % in 20 cycles.

Ta-doped Li[Ni_0.90_Co_0.09_ Ta_0.01_]O_2_: The material is tested in Li metal coin cells at 30 ℃, with 1.2 M LiPF_6_ in EC/DMC electrolyte with 2 wt% vinylene carbonate. The weight ratio of active material in cathode is 90%, and the mass loading is 4-5 mg cm^−2^. The work window is 2.7-4.3V. The highest discharge capacity is achieved in the first cycle, and the capacity retention is 93% in 100 cycles.

Li_5_FeO_4_: The material is tested in Li metal coin cells, with 1.2 M LiPF_6_ in EC/DMC electrolyte. The weight ratio of active material in cathode is 80%. The work window is 1.0-3.8V. The highest discharge capacity is achieved in the first cycle, and the capacity retention is ~89% in 20 cycles.

CD-LNMO: The material is tested in Li metal coin cells, with 0.18 mL 1.0 M LiPF_6_ in EC/DMC electrolyte. The weight ratio of active material in cathode is 75%. The work window is 2.0-4.8V. The highest discharge capacity is achieved in the 9^th^ cycle, and the capacity retention is 71.5% in 80 cycles.

# Supplementary Note 1. Synthesis and characterization of CD-LNMO.

The in situ high-temperature XRD (HTXRD) measurement is performed to probe the atomic reordering of Li_1.46_Ni_0.32_Mn_1.2_O_4-_*_x_* upon calcination. As shown in Supplementary Figure 2, the reflection from proton exchange region^13,14^ (~19°) vanishes at >200 °C and the reflections of 3D spinel-type ordering emerge at >500°C. The ordering of LiTM_6_ superstructure gradually reduces with rising temperature, which is affected by both the removal of proton ions and the atomic reordering. We can then conclude that the heat treatment promotes the reordering of cations to form spinel-type stacking. It is known that the phase transition from layered to spinel structure is triggered by the migration and the reordering of cations.^15,16^ Therefore, the 200-500°C heat-treatment is likely to facilitate the formation of an intermediate state, within which at least partial cations have not formed a 3D spinel-type ordering, while the higher-temperature (>500 °C) treated sample has a more complete cation reordering. We further perform the thermogravimetric (TG) test to determine the proper calcination temperature, from which the weight of the proton-treated sample is relatively stable within 400–700°C (Supplementary Figure 3). Consequently, a highly defective Li_1.46_Ni_0.32_Mn_1.2_O_4-_*_x_* phase is obtained at a moderate temperature (500 °C for 1 h. CD-LNMO), and the Li_1.46_Ni_0.32_Mn_1.2_O_4-_*_x_* phase with more thorough cation reordering is obtained at a higher temperature (700 °C for 1 h. CD-LNMO-H). The XRD patterns of as-synthesized CD-LNMO and CD-LNMO-H are compared with LLO and LNMO (Supplementary Figure 4), representing a layered-dominated ordering in CD-LNMO but spinel-dominated ordering in CD-LNMO-H. This indicates that the atomic evolution from layer to spinel is much more complete in CD-LNMO-H, implying a higher degree of cation ordering in CD-LNMO-H than that of CD-LNMO. The TEM images reveal a nanoplate morphology of CD-LNMO (Supplementary Figure 5), which is raised from the extraction of Li^+^ ions from layered-type oxides.^17^

# Supplementary Note 2. Phase constitutions and cation occupancy probed by synchrotron X-ray diffraction (SXRD) and neutron diffraction (ND)

The phase constitutions and the occupancies of cations in fully charged CD-LNMO (CD-LNMO-C4.8) and fully delithiated CD-LNMO (CD-LNMO-D2.0) are detected by combining the SXRD (Supplementary Figure 7) and ND (Figure 2d and e) measurements. The SXRD pattern of CD-LNMO-C4.8 and CD-LNMO-D2.0 could be fitted well by the partial cation-disordered spinel model (Supplementary Figure 7), while the incorporation of potential secondary phases (Li-rich layered phase: Li_1+_*_x_*TM_1-_*_x_*O_2_ and conventional layered phase: LiTMO_2_ in the charged sample, T1 phase with the space group of I 41/*amd* in discharged sample) could barely improve the refinement (Supplementary Figures 8 and 9). Moreover, all the refinements under the two-phase assumption result in a low concentration of secondary phases (<1%), suggesting the pure phase constitution in the charged and discharged samples.

For the cation occupancies, it should be noted that the scattering factor (*f*) of Li is negligible under X-ray measurements (*f*_Li_ < 2.0), while the neutron scattering lengths of Mn and Ni for ND measurements are -3.73 and 10.3, respectively. Therefore, the occupancy of Li could barely influence the SXRD pattern, and the TM ions disordering within 16d and 16c sites have limited influence on the ND pattern (Supplementary Figure 13). Herein, the occupancy of Mn/Ni is obtained from SXRD results, and the occupancy of Li is probed by ND measurements. It is found that the TM ion disordering within the 16d and 16c in *Fd* *m* spinel phase will result in a structure extinction of (111) reflection,^18^ and the intensity of (111) reflection is substantially affected by the degree of TM ion disordering (Supplementary Figure 10). In our SXRD results, both the CD-LNMO-C4.8 and CD-LNMO-D2.0 represents a weakened (111) reflection compared to that of conventional LNMO, and the refinement reveals ~15% and ~19% of TM ions located in the 16c site under 4.8 V and 2.0 V, respectively (Supplementary Table 2). Hence, we can conclude that the cycled CD-LNMO is a partially-disordered spinel phase. In the ND results, we find a weakening of (004) reflection after the lithiation, probably associated with Li ions insertion. We simulate and compare the ND patterns of CD-LNMO with Li ions located in tetrahedral and octahedral sites (Supplementary Figure 14), to find that the insertion of Li into octahedral sites (16c and 16d) results in a reduced intensity of (004) than that in fully delithiated one, while the insertion of Li into tetrahedral sites (8a) give rise to the increased intensity of (004). This is indicative of a systematic extinction of (004) reflection caused by the octahedral sites-occupancy of Li. The intensity ratio of the (004) and (222) reflections (I_(004)/(222)_) are therefore compared in Supplementary Table 3, confirming that Li ions are preferentially occupied in octahedral sites of fully lithiated CD-LNMO. This is further confirmed by the ND refinements, which reveal negligible Li ions in the 4.8 V charged sample, while most Li ions are inserted into the octahedral sites in the 2.0 V discharged sample (Supplementary Table 2). Based on the refinements, we observed a partial occupancy of 16c sites (74.8%) and 8a sites (19.1%), which may bring some LiO_4_ and LiO_6_ face-sharing. Such ligand face-sharing is of course unstable for transition metal ions with a high TM-TM Coulomb repulsion, but energetically permissible for Li ions.^19^ Therefore, the face-sharing of LiO_4_ and LiO_6_ has been constantly reported in Li-rich compounds.^18-20^

# Supplementary Note 3. Detailed computational methods on constructing the structures

The structure of layered-type Li*_x_*Ni_0.25_Mn_0.75_O_2_ and spinel-type Li*_x_*Ni_0.25_Mn_0.75_O_2_ are obtained from the Materials project, and the disordered rocksalt-type LiNi_0.25_Mn_0.75_O_2_ are obtained through a special quasi-random structure (SQS) method. The SQS calculations are carried out with 80 cation sites/80 anion sites, while pair range up to 7.1 Å, triplet range up to 4.1 Å and quadruplet range up to 4.1 Å are adopted to achieve highly disordering. The structure of Li_1.2_Ni_0.2_Mn_0.6_O_2_ is adopted from the previous reports,^21^ and the cation disordered configuration with the lowest energy among all the probable TM/Li mixed structures is utilized for further analyses.

The structure of LiNi_0.5_Mn_1.5_O_4_ phase (space group: *Fdm*) with uniform Ni/Mn distribution is adopted from the previous reports.^22^ The Li-rich spinel structure and the probable cation disordering are obtained by choosing the configurations with the lowest energy among all the probable Li substitution and Li/cation mixing. To analysis the site energies of Li_oct_ and Li_tet_ in each structure, the supercells with one Li ion located in the 8a or 16c site are constructed for further comparison. For each system, all the 8a and 16c sites are considered, and the site energy differences are obtained from the following equation:

Δ*E*=*E*(Li_oct_)–*E*(Li_tet_)

where *E*(Li_oct_) and *E*(Li_tet_) are the lowest energies of configurations after one Li ions inserted into Li_oct_ and Li_tet_ sites, respectively.

# References

1. House, R. A. et al. Superstructure control of first-cycle voltage hysteresis in oxygen-redox cathodes. *Nature* **577**, 502-508 (2020).

2. Lee, J. et al. Reversible Mn^2+^/Mn^4+^ double redox in lithium-excess cathode materials. *Nature* **556**, 185-190 (2018).

3. House, R. A. et al. Lithium manganese oxyfluoride as a new cathode material exhibiting oxygen redox. *Energy Environ. Sci* **11**, 926-932 (2018).

4. Choi, C. et al. Achieving high energy density and high power density with pseudocapacitive materials. *Nat. Rev. Mater.* **5**, 5-19 (2019).

5. Huang, J. et al. Non-topotactic reactions enable high rate capability in Li-rich cathode materials. *Nature Energy* **6**, 706-714 (2021).

6. Eum, D. et al. Voltage decay and redox asymmetry mitigation by reversible cation migration in lithium-rich layered oxide electrodes. *Nat. Mater* **19**, 419-427 (2020).

7. Yabuuchi, N. et al. High-capacity electrode materials for rechargeable lithium batteries: Li_3_NbO_4_-based system with cation-disordered rocksalt structure. *PNAS* **112**, 7650-7655 (2015).

8. Freire, M. et al. A new active Li-Mn-O compound for high energy density Li-ion batteries. *Nat. Mater* **15**, 173-177 (2016).

9. Kwon, D.-H. et al. The Impact of Surface Structure Transformations on the Performance of Li-Excess Cation-Disordered Rocksalt Cathodes. *Cell Reports Physical Science* **1** (2020).

10. Ji, H. et al. Hidden structural and chemical order controls lithium transport in cation-disordered oxides for rechargeable batteries. *Nat Commun* **10**, 592 (2019).

11. Kim, U.-H. et al. Heuristic solution for achieving long-term cycle stability for Ni-rich layered cathodes at full depth of discharge. *Nat. Energy* **5**, 860-869 (2020).

12. Zhan, C. et al. Enabling the high capacity of lithium-rich anti-fluorite lithium iron oxide by simultaneous anionic and cationic redox. *Nat. Energy* **2**, 963-971 (2017).

13. Paik, Y. et al. Lithium and Deuterium NMR Studies of Acid-Leached Layered Lithium Manganese Oxides. *Chem. Mater* **14**, 5109-5115 (2002).

14. Cheng, F., Chen, J., Zhou, H. & Manthiram, A. Structural and Electrochemical Characterization of (NH_4_)_2_HPO_4_-Treated Lithium-Rich Layered Li_1.2_Ni_0.2_Mn_0.6_O_2_ Cathodes for Lithium-Ion Batteries. *J. Electrochem. Soc* **160**, A1661-A1667 (2013).

15. Mohanty, D. et al. Unraveling the Voltage-Fade Mechanism in High-Energy-Density Lithium-Ion Batteries: Origin of the Tetrahedral Cations for Spinel Conversion. *Chem. Mater* **26**, 6272-6280 (2014).

16. Chen, Q. et al. Highly reversible oxygen redox in layered compounds enabled by surface polyanions. *Nat. Commun* **11**, 3411 (2020).

17. Tang, W. Preparation of Plate-Form Manganese Oxide by Selective Lithium Extraction from Monoclinic Li_2_MnO_3_ under Hydrothermal Conditions. *Chem. Mater* **12,**, 3271-3279 (2000 ).

18. Cai, Z. et al. Realizing continuous cation order-to-disorder tuning in a class of high-energy spinel-type Li-ion cathodes. *Matter* **4**, 1-20 (2021).

19. Liu, H. et al. A disordered rock salt anode for fast-charging lithium-ion batteries. *Nature* **585**, 63-67 (2020).

20. Ji, H. et al. Ultrahigh power and energy density in partially ordered lithium-ion cathode materials. *Nat. Energy* **5**, 213-221 (2020).

21. Li, X. et al. Direct Visualization of the Reversible O^2-^/O^-^ Redox Process in Li-Rich Cathode Materials. *Adv. Mater* **30**, 1705197 (2018).

22. Shiiba, H. et al. Defect Formation Energy in Spinel LiNi_0.5_Mn_1.5_O_4-δ_ Using Ab Initio DFT Calculations. *J. Phys. Chem. C* **119**, 9117-9124 (2015).
