# Supplementary material for: A medium-entropy transition metal oxide cathode for high-capacity lithium metal batteries
Source: Nat Commun. 2022 Oct 18;13:6158. doi: 10.1038/s41467-022-33927-0 (PMC9579144; doi:10.1038/s41467-022-33927-0)
Supplement: Supplementary file 2 — Reporting Summary [file 41467_2022_33927_MOESM2_ESM.pdf]

nature portfolio

Corresponding author(s): Cheng-yan Xu
Last updated by author(s): Sep 16, 2022

Reporting Summary

Nature Portfolio wishes to improve the reproducibility of the work that we publish. This form provides structure for consistency and transparency in reporting. For further information on Nature Portfolio policies, see our Editorial Policies and the Editorial Policy Checklist.

Statistics

For all statistical analyses, confirm that the following items are present in the figure legend, table legend, main text, or Methods section.

n/a	Confirmed	
☐	☒	The exact sample size (n) for each experimental group/condition, given as a discrete number and unit of measurement
☐	☒	A statement on whether measurements were taken from distinct samples or whether the same sample was measured repeatedly
☐	☒	The statistical test(s) used AND whether they are one- or two-sided<br>*Only common tests should be described solely by name; describe more complex techniques in the Methods section.*
☐	☒	A description of all covariates tested
☐	☒	A description of any assumptions or corrections, such as tests of normality and adjustment for multiple comparisons
☐	☒	A full description of the statistical parameters including central tendency (e.g. means) or other basic estimates (e.g. regression coefficient) AND variation (e.g. standard deviation) or associated estimates of uncertainty (e.g. confidence intervals)
☐	☒	For null hypothesis testing, the test statistic (e.g. F, t, r) with confidence intervals, effect sizes, degrees of freedom and P value noted<br>*Give P values as exact values whenever suitable.*
☐	☒	For Bayesian analysis, information on the choice of priors and Markov chain Monte Carlo settings
☐	☒	For hierarchical and complex designs, identification of the appropriate level for tests and full reporting of outcomes
☐	☒	Estimates of effect sizes (e.g. Cohen's d, Pearson's r), indicating how they were calculated

Our web collection on statistics for biologists contains articles on many of the points above.

Software and code

Policy information about availability of computer code

Data collection	no code used in this paper
Data analysis	no code used in this paper

For manuscripts utilizing custom algorithms or software that are central to the research but not yet described in published literature, software must be made available to editors and reviewers. We strongly encourage code deposition in a community repository (e.g. GitHub). See the Nature Portfolio guidelines for submitting code & software for further information.

Data

Policy information about availability of data

All manuscripts must include a data availability statement. This statement should provide the following information, where applicable:

- Accession codes, unique identifiers, or web links for publicly available datasets
- A description of any restrictions on data availability
- For clinical datasets or third party data, please ensure that the statement adheres to our policy

The data generated in this study are provided in Source data. Extra data that support the findings of this study are available from the corresponding authors upon reasonable request.

Human research participants

Policy information about studies involving human research participants and Sex and Gender in Research.

Reporting on sex and gender	No human research involved in this paper
Population characteristics	No human research involved in this paper
Recruitment	No human research involved in this paper
Ethics oversight	No human research involved in this paper

Note that full information on the approval of the study protocol must also be provided in the manuscript.

Field-specific reporting

Please select the one below that is the best fit for your research. If you are not sure, read the appropriate sections before making your selection.

☐ Life sciences ☐ Behavioural & social sciences ☒ Ecological, evolutionary & environmental sciences

For a reference copy of the document with all sections, see nature.com/documents/nr-reporting-summary-flat.pdf

Ecological, evolutionary & environmental sciences study design

All studies must disclose on these points even when the disclosure is negative.

Study description	This work reveal a novel method to prepare a cation disordered spinel cathode materials, and this material show a high reversible capacity (314.1 mA h g-1)
Research sample	All the research sample are synthesized through mentioned method in the method section. The sample is optimized by changing the calculation temperature, which is compared in the paper.
Sampling strategy	We have not do the sample statistics in this work
Data collection	The XAS data is collected in Beijing Synchrotron Radiation Facility (BSRF), Soochow Beamline for Energy Materials,Advanced Light Source (ALS), Lawrence Berkeley National Laboratory (LBNL) by pengjun zhang, yang ha, wanli yang, li song with the method in method section. The SXRD data is collected in Beijing Synchrotron Radiation Facility (BSRF) by Yu Chen with the method in method section. The ND data is collected in China Spallation Neutron Source (CSNS) by qingyong ren, wen yin and xin tong with method in method section. The TEM data is collected in Nanjing University by meiyu wang and peng wang with the method in method section. The electrochemical, TGA, in-situ XRD , SEM and DFT data is collected by yi pei, qing chen and chengyan xu in Harbin Institute of Technology with the method in method section.
Timing and spatial scale	All the data range are shown in the figures, and the data reduction ranges, including the k range of EXAFS are shown in the method section. No gap is used in the data collection.
Data exclusions	No data are excluded in the analysis.
Reproducibility	The synthesize of sample has been repeated several times for the electrochemical, phase, in-situ and ex-situ characterizations. All the characterizations consistently show the same results that convince the repeatability.
Randomization	No randomization is required in this work.
Blinding	No blinding is required in this work.

Did the study involve field work? ☐ Yes ☒ No

Reporting for specific materials, systems and methods

We require information from authors about some types of materials, experimental systems and methods used in many studies. Here, indicate whether each material, system or method listed is relevant to your study. If you are not sure if a list item applies to your research, read the appropriate section before selecting a response.

Materials & experimental systems

n/a	Involved in the study
☒	☐ Antibodies
☒	☐ Eukaryotic cell lines
☒	☐ Palaeontology and archaeology
☒	☐ Animals and other organisms
☒	☐ Clinical data
☒	☐ Dual use research of concern

Methods

n/a	Involved in the study
☒	☐ ChIP-seq
☒	☐ Flow cytometry
☒	☐ MRI-based neuroimaging
